# Size, microhabitat, and loss of larval feeding drive cranial diversification in frogs

**DOI:** 10.1038/s41467-021-22792-y

**Published:** 2021-05-04

**Authors:** Carla Bardua, Anne-Claire Fabre, Julien Clavel, Margot Bon, Kalpana Das, Edward L. Stanley, David C. Blackburn, Anjali Goswami

**Affiliations:** 1 Department of Life Sciences, Natural History Museum, London, UK; 2 Department of Genetics, Evolution & Environment, University College London, London, UK; 3 Paläontologisches Institut und Museum, Universität Zürich, Zürich, Switzerland; 4 Univ Lyon, Université Claude Bernard Lyon 1, CNRS, ENTPE, UMR 5023 LEHNA, F-69622, Villeurbanne, France; 5 Museum für Naturkunde, Leibniz Institut für Evolutions und Biodiversitätsforschung, Berlin, Germany; 6 Department of Natural History, Florida Museum of Natural History, University of Florida, Gainesville, FL USA

**Keywords:** Evolutionary ecology, Evolutionary developmental biology, Phylogenetics, Herpetology

## Abstract

Habitat is one of the most important factors shaping organismal morphology, but it may vary across life history stages. Ontogenetic shifts in ecology may introduce antagonistic selection that constrains adult phenotype, particularly with ecologically distinct developmental phases such as the free-living, feeding larval stage of many frogs (Lissamphibia: Anura). We test the relative influences of developmental and ecological factors on the diversification of adult skull morphology with a detailed analysis of 15 individual cranial regions across 173 anuran species, representing every extant family. Skull size, adult microhabitat, larval feeding, and ossification timing are all significant factors shaping aspects of cranial evolution in frogs, with late-ossifying elements showing the greatest disparity and fastest evolutionary rates. Size and microhabitat show the strongest effects on cranial shape, and we identify a “large size-wide skull” pattern of anuran, and possibly amphibian, evolutionary allometry. Fossorial and aquatic microhabitats occupy distinct regions of morphospace and display fast evolution and high disparity. Taxa with and without feeding larvae do not notably differ in cranial morphology. However, loss of an actively feeding larval stage is associated with higher evolutionary rates and disparity, suggesting that functional pressures experienced earlier in ontogeny significantly impact adult morphological evolution.

## Abstract (web summary)

The evolution of metamorphic species may be constrained by different ecologies of the larval and adult stages. Here, Bardua et al. show that in frogs, adult ecology is more important than larval ecology for skull evolution, but species that don’t feed as tadpoles evolve faster than those that do.

## Introduction

Adaptations to habitats are responsible for some of the most striking examples of phenotypic evolution, driving profound shifts in morphology^1–6^, in shape diversity (‘disparity’)^7^ and in rate of shape evolution^1,8–11^. Adaptations to similar habitats also result in phenotypic convergence when responses to selection produce similar shifts in phenotypic optima across diverse clades^2,12–15^, as in the body forms of pelagic fishes, ichthyosaurs and whales. Habitat exploitation clearly plays a major role in shaping morphological evolution and macroevolutionary dynamics across organisms. However, an organism’s ability to specialise for an adult habitat may be constrained by adaptations to conditions experienced at earlier developmental stages. Species with morphologically and ecologically distinct developmental stages may experience confounding and competing selective pressures throughout ontogeny which may limit their ability to evolve specific morphologies^16–18^. The potential constraints of divergent selection at different developmental stages may drive selection for a simpler life history, i.e. one with no abrupt ontogenetic change in morphology or niche. Accordingly, the loss of a free-living, actively feeding larval stage has been credited with promoting morphological novelty^19,20^ and driving increased rates of morphological evolution across salamanders and caecilians^11,21,22^. Decoupling adult and embryonic form via an ecologically distinct larval stage may circumvent this potentially antagonistic selection by creating developmental modules that allow adult phenotype to evolve independently of the constraints of earlier life stages^23–25^. Investigating the relative influences of adult ecology and potential constraints of ontogenetic form on morphological evolution is complex given that the two are inherently linked. Developmental changes may act as exaptations that promote the exploitation of a habitat^26^, or, alternatively, adaptation to environment may drive shifts in developmental strategy^27^. Furthermore, both factors likely interact with other traits, such as body size or developmental timing^28,29^, and their influence may vary across morphological structures. Untangling the roles of ecology and development on morphological diversification thus necessitates a macroevolutionary and multifaceted approach.

With more than 7300 extant species, frogs (Lissamphibia: Anura) are one of the most diverse orders of vertebrates and show remarkable variation in both life history and ecology. Although frogs are predominantly generalist predators, they are ecologically diverse, including species specialised for fossorial, aquatic, arboreal and terrestrial microhabitats^30–32^, which has facilitated their widespread and near-global distribution^33^. Because anurans vary widely in body size, ranging from a snout–vent lengths of ~7 to 320 mm, there are ample opportunities for exploring the relationship between microhabitat specialisation and body size, which across organisms affects many ecological traits^34^, and the evolution of shape^35^. Life history strategies in frogs range from direct development (without a free-living, feeding larval phase) to fully biphasic, with free-living feeding larval phases (i.e. tadpoles) specialised to a wide range of aquatic microhabitats and diets that differ from adult frogs. Biphasic frogs undergo radical morphological changes during metamorphosis; the magnitude of ontogenetic change is less in direct-developing species^36,37^ though the extent of reorganisation of internal anatomy remains poorly explored. All frogs thus experience some degree of morphological transformations through ontogeny, but species vary extensively in the degree of interaction with their environment as larvae. As feeding is one of the most critical functions of the cranium, larval feeding likely imposes strong selection pressures on the developing cranium. In particular, taxa with feeding larvae require distinct functional adaptations for their larval and adult environments, given the distinct changes in feeding mechanisms across the two life stages^33,38–40^. For this reason, the presence of an actively feeding larval stage has been hypothesised to be an ontogenetic constraint on the adult morphology, whereas the loss of an actively feeding larval stage has been suggested to promote morphological novelty^19,20,41,42^ and drive increased rates of morphological evolution^11,21,22^. This ontogenetic transition in function may also differentially impact structures based on their relative timing of development, for example with greater constraints on early-ossifying bones^43^.

The developmental and ecological complexity of frogs provide an ideal opportunity to test competing hypotheses on the relative influences of ecological and developmental factors on the diversification of frog morphology. Some previous studies have addressed how larval morphology impacts adult morphology in frogs, primarily with data on body size^44–46^, which is easily comparable across stages and taxa, or with studies focused on one or a few anuran families^44,47,48^. While there is a correlation between larval and adult body size across all frogs^45^, the strength of this relationship varies across families^44^, and there is no significant correlation in rates of evolution of adult and larval size^45^. Studies capturing aspects of morphology other than body size support decoupling of larval and adult evolution but are limited both in taxonomic breadth and morphological data^47,48^. Despite long-standing hypotheses of decoupled evolution between life history stages and its importance to understanding the prevalence of complex life cycles in animals, there are no studies that test this hypothesis with data that captures both the complexity of adult morphology and its diversity across frogs. Moreover, no studies have explicitly compared the relative influence of developmental effects to other factors, such as adult ecology. If the anuran larval stage acts as a developmental module that fully decouples larval and adult phenotypic evolution, then we would expect no differences in adult morphological evolution between taxa with different life history strategies. If so, then we would expect factors such as adult microhabitat to be more significant influences on adult morphological evolution. In contrast, if larval and adult evolution is correlated, then the compound effects of divergent selection pressures acting on larval and adult stages are expected to influence, and potentially constrain, phenotypic evolution of the adult form in taxa with feeding larvae. Furthermore, developmental and adult ecological factors may interact, meaning that both adult microhabitat and adult morphological evolution might be influenced by the degree to which larval forms interact with their environment.

**Fig. 1:**
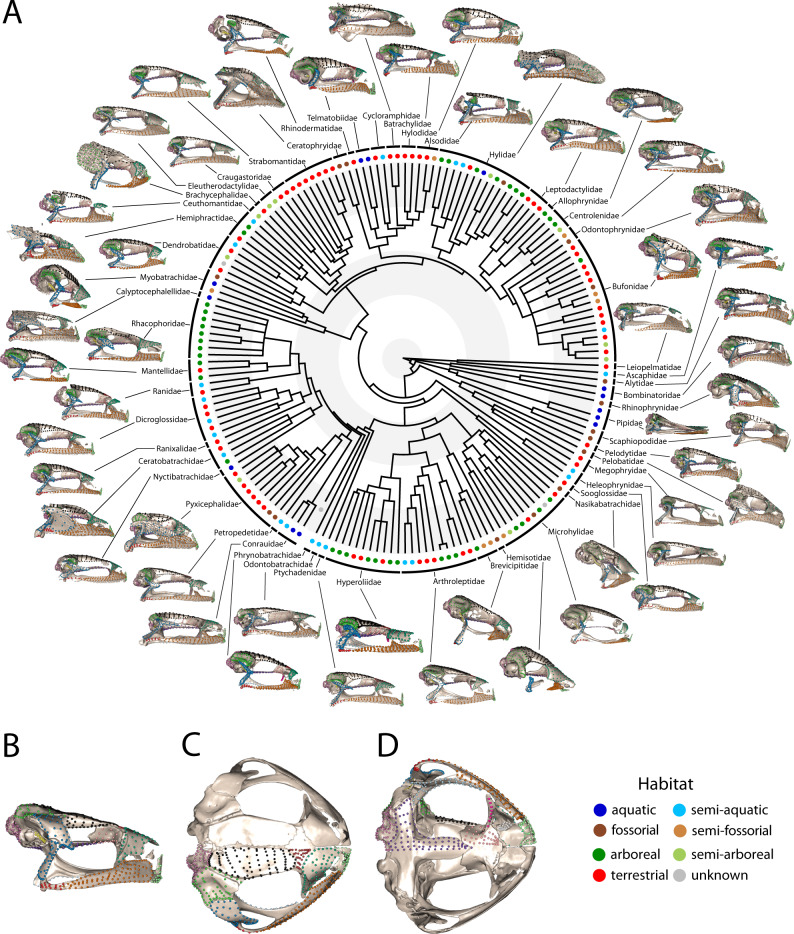
Cranial shape diversity across Anura. **A** Defined cranial regions displayed on one anuran skull from every family across the phylogeny (lateral aspect, skulls not to scale). Phylogeny for the 173 species in this study modified from Jetz and Pyron^113^ with the addition of *Thaumastosaurus gezei*. Branch lengths are scaled to time, with alternate shaded bands indicating periods of 50 million years (outer extreme at Recent). For details on specimens visualised here, see Supplementary Data 1. Landmarks and semilandmarks in **B** lateral, **C** dorsal and **D** ventral views, shown on *Adenomus kelaartii* (FMNH:Amphibians and Reptiles:1580). Points are coloured according to the 15 regions defined in this study. Regions are as follows: Occ (light purple) occipital region, FP (black) frontoparietal, Qj (red) quadratojugal, Max (orange) maxilla, Na (green) nasal, Neo (hot pink) Neopalatine, Otic (lime green) otic region, Pm (pale green) premaxilla, PS (purple) parasphenoid, Pt (light blue) pterygoid, SphD (brown) sphenethmoid (dorsal surface), SphV (light pink) sphenethmoid (ventral surface), Sq (blue) squamosal, St (yellow) stapes, Vo (grey) vomer.

Given the extensive shape variation^49,50^ and the potential heterogeneity of ecological and developmental influences across cranial regions, the adult skull provides an ideal system for testing these hypotheses. As in all vertebrates, the anuran cranium comprises multiple osteological units with different functions, embryonic origins and types and timings of ossification^43,51–53^. Anurans have the added complexity that many cranial bones are variably present between species^50,54,55^, and their crania likely exhibit substantial mosaic evolution^56–63^. To tackle this challenge, we quantify anuran cranial complexity using a high-density surface morphometric approach that represents a significant advance over previous work which used a limited number of landmarks and excluded structures that are variably present, including a number of key functional elements such as the neopalatine, sphenethmoid, vomer, quadratojugal and stapes^3,64^. With these data, we reconstruct the morphological evolution of the anuran skull, spanning the full range of frog ecological, developmental and morphological diversity (Fig. 1). We estimate the relative influences and interactions of adult microhabitat, skull size, larval feeding, and ossification timing on the morphology and diversification of the frog cranium and provide an empirical analysis of the larval-adult decoupling hypothesis using 3D shape data across the full phylogenetic breadth of frogs.

In this work, we show that skull size, adult microhabitat, larval feeding and ossification timing are all significant factors shaping aspects of cranial evolution in frogs, with late-ossifying elements showing the greatest disparity and fastest evolutionary rates. We identify a ‘large size-wide skull' pattern of anuran, and possibly amphibian, cranial evolutionary allometry driven by structural constraints and feeding adaptations. Skull size and microhabitat consistently show the strongest effects on cranial shape, with fossorial, semi-fossorial and aquatic adult microhabitats occupying distinct regions of morphospace and displaying increased rates of evolution and high disparity, likely reflect specialisations of adult feeding and hearing functions. Taxa with and without feeding larvae do not notably differ in their cranial morphology, supporting partial adult-larval decoupling. However, loss of an actively feeding larval stage is associated with higher rates of evolution and disparity in most cranial regions, suggesting that functional pressures experienced earlier in ontogeny still significantly impact patterns of adult morphological evolution in frogs. Our analysis thus provides a comprehensive assessment of the relative effects of components of larval and adult ecology, and their interaction, on the morphological evolution of frogs, with relevance for understanding the evolution, and consequences, of complex life cycles across the tree of life.

## Results

### Cranial shape

**Fig. 2:**
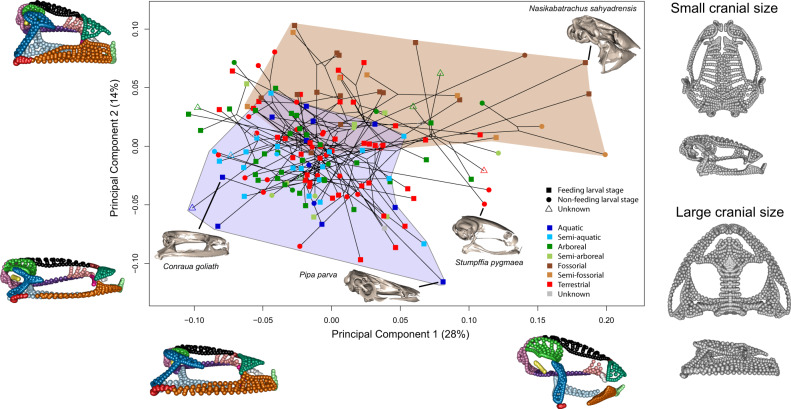
Phylomorphospace of anuran skull variation. Each specimen’s location is coloured by microhabitat and the symbols indicate larval feeding. The maximum morphospace occupation for fossorial (including semi-fossorial) and aquatic (including semi-aquatic) species is illustrated by shading in the respective regions brown and blue. Specimen meshes are included to demonstrate the general shape differences between fossorial (*Nasikabatrachus sahyadrensis*) and aquatic (*Pipa parva*) species, as well as the specimens with the largest (*Conraua goliath*) and smallest (*Stumpffia pygmaea*) crania. Extreme shapes along both axes are visualised (in lateral aspect) by deforming the shape data along each axis and are coloured by cranial region (see Supplementary Fig. 1). Estimated cranial morphology at minimum and maximum cranial size is also presented, shown in dorsal (top) and lateral (bottom) aspects. Landmark data were mirrored for visualisation purposes only.

Principal components (PCs) analysis of the entire adult cranium (Fig. 2 and Supplementary Table 1) showed that the predominant shape changes along PC1 (28% of variance) were the height of the cranium, the anteroposterior translation of the quadratojugal, the lateral expansion of the frontoparietal, the reduction of the maxillary arcade and the shortening of the anterior process of the squamosal (Supplementary Note 1, Supplementary Figs. 1, 2 and see Fig. 2 for the cranial regions). PC2 (14%) represented variation associated with the height of the maxilla, a larger nasal, and an anteroposteriorly compressed frontoparietal, as well as a taller, wider cranium (Supplementary Fig. 3).

The first two PCs separated fossorial (including semi-fossorial) and aquatic (including semi-aquatic) taxa, whereas terrestrial, arboreal and semi-arboreal species overlapped with both (Fig. 2). Taxa with and without an actively feeding larval stage overlap entirely in cranial morphospace (Supplementary Fig. 4). Phylogenetic regression of cranial shape on centroid size, microhabitat and larval feeding supported all three factors as significantly associated (*p* < 0.01) with shape of the entire skull and most cranial elements (Table 1 and Supplementary Table 2). Presence of an actively feeding larval stage consistently displayed the lowest effect size of the three factors, was not significantly associated with the shape of the nasal, stapes or dorsal sphenethmoid, and was only marginally significant (0.05 > *p* > 0.01) for the otic, maxilla and quadratojugal. Skull size and adult microhabitat showed similar effect sizes for most elements and were significantly associated with shape of all elements, with the exception of adult microhabitat showing no significant association with the neopalatine and only a marginally significant one with the dorsal sphenethmoid (Table 1).

**Table 1:** Region-specific analyses of shape.

Cranial region	Disparity (Procrustes variance) (× 10^−5^)	Evolutionary rate, σ^2^*mult*, (× 10^−9^)	Ossification sequence rank	Evolutionary allometry (SES)	Habitat (SES)	Larval feeding (SES)
Frontoparietal	1.04	0.349	2	7.85***	8.17***	4.06***
Maxilla	1.35	3.56	6	5.67***	7.22***	2.13*
Nasal	1.07	1.91	7	7.21***	7.77***	0.30
Neopalatine	1.91	34.8	12	5.47***	1.01	2.72**
Occipital	0.72	2.39	3	7.50***	8.34***	3.72***
Otic	1.13	3.87	4	8.18***	5.66***	1.67*
Parasphenoid	0.60	3.84	1	7.51***	7.01***	3.49***
Premaxilla	0.89	5.16	5	5.41***	7.57***	3.30**
Pterygoid	1.20	7.86	10	7.06***	6.27***	3.19***
Quadratojugal	2.92	27.8	11	9.21***	6.81***	2.31*
Sphenethmoid (dorsal)	1.29	12.7	13	5.66***	2.10*	0.08
Sphenethmoid (ventral)	1.96	2.33	13	8.38***	7.28***	3.67**
Squamosal	1.81	5.03	8	6.91***	9.92***	3.63***
Stapes	1.86	17.5	NA	10.84***	12.65***	1.35
Vomer	0.99	5.37	9	7.78***	4.53***	3.83***

Disparity is measured as Procrustes variance scaled by semi/landmark number. Evolutionary rate estimates include error. Median ossification sequence rank taken from Weisbecker^43^. Effect sizes (SES) and significances for evolutionary allometry (size), microhabitat and larval feeding from Type II phylogenetic regressions of cranial shape with all three predictors and all interactions using the MCC tree. All tests are one-sided, with significances based on permutations (*n* = 999). Interactions, Pillai’s test results, Pagel’s lambda and results for sample of 100 trees are detailed in Supplementary Table 2. Significance of results for evolutionary allometry, influence of microhabitat, and influence of larval feeding is as follows: *p* values significant at the following alpha levels: *≤0.05, **≤0.01, ***≤0.001.

There were significant interactions among size, adult microhabitat and larval feeding for the entire skull and most elements except for the nasal, neopalatine and dorsal sphenethmoid (*p* > 0.05) and marginally significant interactions (0.05 > *p* > 0.01) for the parasphenoid, premaxilla and quadratojugal (Supplementary Table 2). Size and adult microhabitat had highly significant interactions (*p* < 0.01) for most elements (Supplementary Fig. 4), as did adult microhabitat and larval feeding (Supplementary Fig. 5), whereas size and larval feeding showed a highly significant interaction in only six elements. Replicating these analyses across a distribution of 100 phylogenetic trees demonstrates that these results are also robust to phylogenetic uncertainty (Supplementary Table 2).

Given the significant influence of size on cranial shape and the significant interaction of microhabitat with size for most cranial elements, we further investigated the variation associated with change in skull size across anurans. Larger species had wider, more triangularly-shaped skulls with relatively smaller cranial vaults (i.e. frontoparietal and parasphenoid) and occipital regions (Fig. 1) and a more horizontally-oriented suspensory apparatus (pterygoid, squamosal and quadratojugal). Aquatic species had larger crania than taxa from other microhabitats (Supplementary Fig. 4 and Supplementary Note 1) although size ranges overlapped across all microhabitats. Removing allometric size from shape data prior to morphospace analysis increased the separation of fossorial and aquatic taxa (Supplementary Fig. 6). Shape variation captured by the first four PC axes was similar for both the original and size-corrected shape data (Supplementary Fig. 7), with the main difference being increased shift in the lateral extension of the maxilla and otic regions on PC1 for the size-corrected data.

### Ancestral shape and trait estimation

**Fig. 3:**
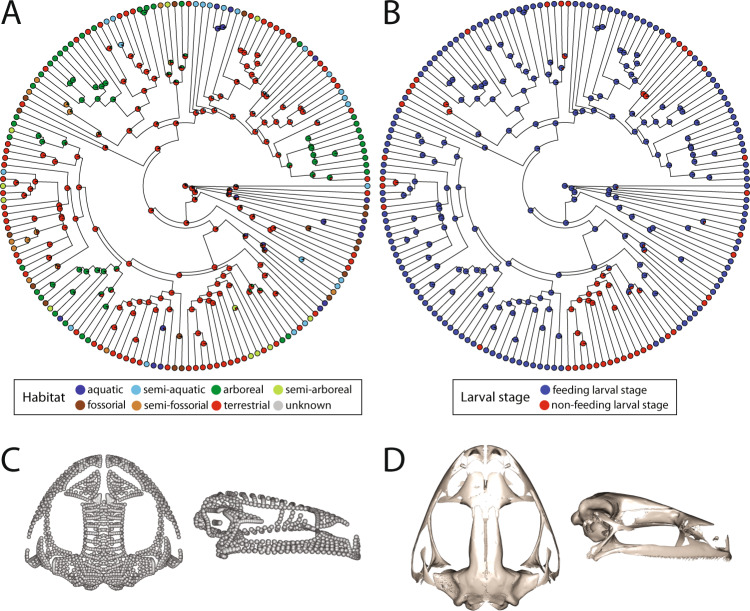
Ancestral state and shape reconstructions. **A** Ancestral state estimation for microhabitat (Total *N* for microhabitat = 170, three specimens with missing microhabitat data, as described in Methods). **B** Ancestral state estimation for larval feeding (Total *N* for larval feeding = 163, ten specimens with missing larval feeding mode data, as described in Methods). **C** Ancestral shape reconstruction for cranial shape in dorsal (left) and lateral (right) aspect. **D** Specimen cranium closest in shape to the ancestral shape reconstruction (*Minervarya nilagirica*, MNHN:RA:19842337) in dorsal (left) and lateral (right) aspect.

Ancestral state estimation of microhabitat and larval feeding mode supported the ancestral condition for frogs as terrestrial and with actively feeding larvae (Fig. 3). Transitions between all microhabitats are widespread across frogs, and a non-feeding larval stage has arisen at least 15 times among the taxa included in our analysis. A terrestrial ancestor with feeding larvae is consistent with the ancestral reconstruction of anuran cranial shape (Fig. 3), which we estimate as most similar to *Minervarya nilagirica* (which likely is terrestrial with an actively feeding larval stage).

### Evolutionary rate and disparity

**Fig. 4:**
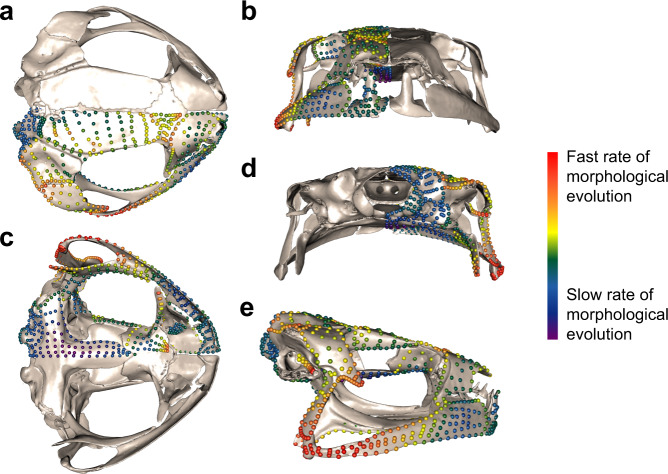
Disparity across the frog cranium. Landmarks and semilandmarks shown on *Adenomus kelaartii* (FMNH: Amphibians and Reptiles: 1580) colour graded by magnitude of disparity (Procrustes variance), from low (purple) to high (red), in **a** dorsal, **b** anterior, **c** ventral, **d** posterior and **e** lateral aspect.

Cranial regions show considerable heterogeneity in evolutionary rate (*σ*^*2*^_mult_) and disparity (Procrustes variance). The highest evolutionary rates and disparity were concentrated in the neopalatine and quadratojugal, reflecting extensive variation in feeding mechanisms that impact the relative position, orientation and extent of ossification of the jaw joint (Table 1 and Fig. 4). The frontoparietal, occipital and nasal evolved slowest and were least disparate, likely reflecting the conserved functions of these regions (e.g. protection of the brain and sensory organs and neck articulation).

**Fig. 5:**
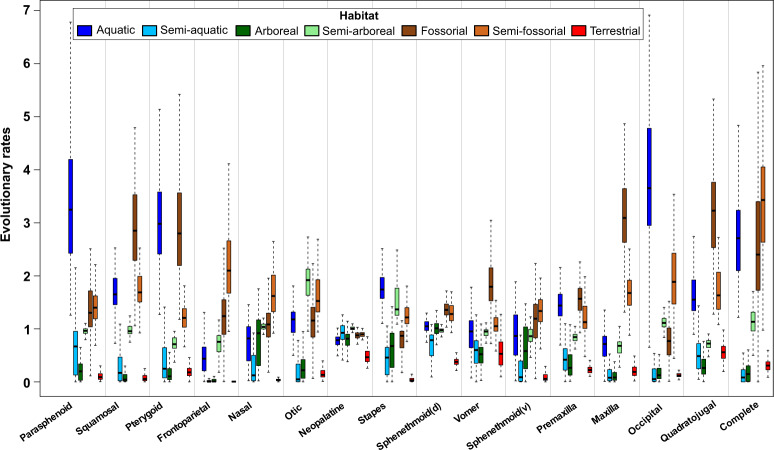
Region-specific rates of evolution for different microhabitats. Evolutionary rate for each of the 15 cranial regions and the cranium across different microhabitats estimated across a random sample of 100 trees drawn in the posterior distribution of Jetz and Pyron^113^. Intraspecific variation and measurement error were jointly estimated along with evolutionary rates during model fit to account for uncertainty in traits values that cmay cause model departure. Rates are standardised by the mean, and box bounds represent the median with the first and third quartile, while whiskers indicate the minimum and maximum values within 1.5x the interquartile range.

When taxa were divided based on adult microhabitat, striking differences in evolutionary rate and disparity were observed for the whole cranium and most cranial regions (Table 1, Fig. 5 and Supplementary Fig. 8). The crania of fossorial, semi-fossorial and aquatic species evolved at the fastest rates (Fig. 5), with semi-aquatic, arboreal and terrestrial species exhibiting the slowest rates. Across individual cranial regions, fossorial, semi-fossorial and aquatic species also exhibited considerably, and, in many comparisons, significantly, faster rates of evolution than the remaining microhabitat specialists, especially for the quadratojugal and squamosal regions (and the stapes for fossorial and semi-fossorial species) (Fig. 5). The whole cranium and most cranial regions of fossorial, semi-fossorial and aquatic species were also significantly more disparate than those of taxa from the remaining microhabitats (Supplementary Fig. 8 and Supplementary Table 3).

No significant difference was found between the rate of cranial evolution for taxa with or without feeding larvae across the whole skull, but taxa with non-feeding larvae showed significantly faster evolutionary rates in eight of the 15 analysed regions: the nasal, otic, neopalatine, stapes, dorsal sphenethmoid, vomer and occipital (Supplementary Fig. 9). Rates of evolution for taxa with non-feeding larvae were also higher for most other cranial regions, although not significantly so (Supplementary Fig. 9 and Supplementary Table 4). Despite representing a minority of species in our dataset (39 non-feeding vs 124 feeding), taxa with non-feeding larvae had higher disparities for the whole cranium (non-feeding: 0.0120, feeding: 0.0095 *p* = 0.135) and most individual regions, although this difference was only significant for four cranial regions (premaxilla, otic, sphenethmoid (ventral) and vomer) and not for the entire cranium (Supplementary Fig. 9 and Supplementary Table 4).

**Fig. 6:**
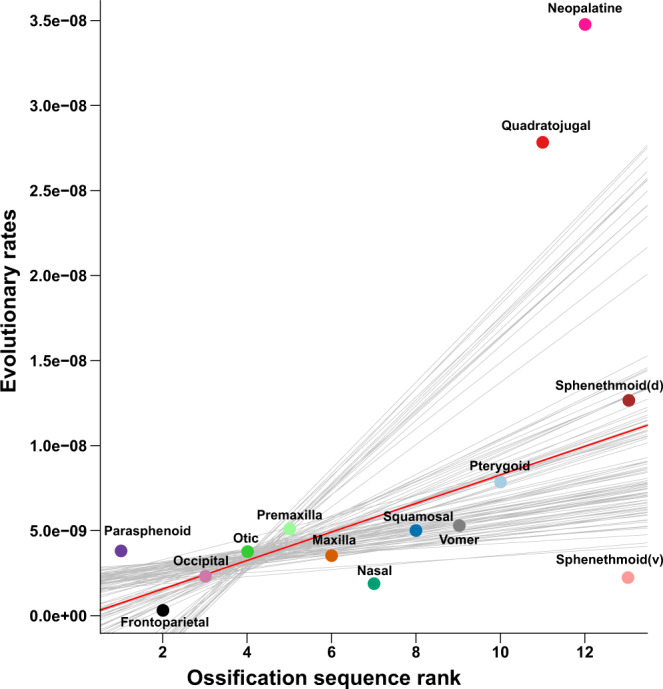
Ossification sequence and evolutionary rate. Ossification sequence rank versus evolutionary rate for each cranial region. Evolutionary rates are estimated with error to account for intraspecific variance and departures from Brownian Motion (results estimated without error in Supplementary Fig. 11), with lines derived from a robust regression fit. Two-sided Spearman’s rank correlation of evolutionary rate with ossification sequence rank (from Weisbecker & Mitgutsch^43^) was significant (mean Spearman’s rho = 0.50, *p* < 0.01). Colours for cranial regions are as indicated in Fig. 1.

Ossification sequence rank (relative timing) was significantly correlated with disparity (*ρ* = 0.76, *p* = 0.002) and evolutionary rate (mean *ρ* = 0.50, *p* < 0.01 with error estimated, mean *ρ* = 0.61, *p* < 0.01 without error), with later-ossifying bones exhibiting greater disparity and faster rates of evolution (Fig. 6 and Supplementary Figs. 10, 11).

## Discussion

Our analyses support reconstructions of the ancestral frog as terrestrial and with an actively feeding larval stage^65^, which was subsequently lost at least 15 times. Given our high-level sampling and focus on one aspect of life history, transitions to derived life history modes, including direct development, are likely even more complex and more widespread in anuran evolution. Transitions in adult microhabitat are also common across frogs, providing an ideal study system for comparing the relative influences of potential selective pressures in larval and adult stages. Our results support microhabitat and skull size as the dominant factors driving cranial shape evolution in frogs, with aquatic, fossorial and semi-fossorial taxa displaying the fastest rates of evolution (Fig. 5), the highest cranial morphological diversity (Supplementary Fig. 8) and distinct cranial morphologies (Fig. 2). Larval feeding mode is also a significant influence on skull shape across the whole skull and most cranial regions, with loss of feeding larvae consistently associated with greater disparity and faster evolutionary rates than those that maintain an actively feeding larval stage.

Adaptation to adult microhabitats has resulted in substantial variation in cranial shape, disparity and rate of evolution across frogs, particularly for aquatic, fossorial and semi-fossorial taxa. Subterranean habitats can minimise exposure to environmental and biotic fluctuations, but are also functionally challenging, creating considerable locomotory and sensory system constraints^66,67^. Similarly, the dense aquatic medium imparts significant functional pressures on locomotion and feeding^68^. These challenging habitats can promote^11,22,62^ or limit^69^ phenotypic evolution, and here we find evidence of the former, likely due to divergent specialised feeding modes within species considered the same microhabitat specialist (e.g. *Pyxicephalus* and *Nasikabatrachus* are both fossorial frogs). Interestingly, the crania of semi-aquatic taxa are the least disparate and slowest evolving, suggesting their more generalist approach does not impose the same functional pressures. Alternatively, semi-aquatic taxa may be limited by the divergent selection pressures of both aquatic and terrestrial microhabitats^70^, an interpretation supported by our findings that semi-aquatic and terrestrial frogs overlap in morphospace and display similar patterns of cranial disparity and evolutionary rate. For the shape of individual elements, those that are most strongly associated with adult microhabitat also show the largest differences in evolutionary rates across microhabitats. Recent work has also demonstrated that adult microhabitat interacts significantly with presence of hyperossification of the skull, which is found in all microhabitat specialists, further supporting adult microhabitat as a key factor influencing many aspects of cranial shape in anurans^64^.

Size is also a major influence on morphology in many clades (e.g.^35^), and our analyses support allometry as a significant component of anuran cranial variation, with cranial width increasing with size. Our results for anurans, in combination with previous studies of caecilians and salamanders^22,64,71^, suggest an amphibian counterpart to the mammalian-based craniofacial evolutionary allometry rule (CREA) that large body size results in long faces^35,72^. Instead, however, large size results in wide skulls^22,64,71^ in amphibian cranial evolutionary allometry (ACREA). This pattern may be driven by both structural constraints and feeding adaptations. Structurally, we find that larger crania have proportionally smaller braincases (congruent with mammals^72^), and the occipital region shows the strongest relationship to size, as found across caecilians^22^. Smaller crania exhibit relatively larger occipital regions, otic regions and cranial vaults, and more vertically-oriented suspensory apparatuses in order to maintain the minimum space requirements for the brain and sensory organs^73^. Thus, size constraints related to the nervous and sensory systems have a greater influence on the morphology of smaller crania. In terms of feeding, frogs are gape-limited predators^74^, and the consumption of large prey requires wide skulls^75^. Functional requirements related to feeding may therefore contribute to the ‘large size-wide skull’ pattern of skull allometry. For example, suction feeding, which is present in some aquatic species, requires a wider mouth^76–78^, supporting a link between cranial size, feeding function and microhabitat. Relatedly, we recovered a highly significant interaction between microhabitat and cranial size across the skull and in most cranial regions, with aquatic taxa exhibiting larger crania.

Feeding mechanics appears to be a considerable driving force behind the cranial evolution of frogs. The jaw articulation region of frogs (quadratojugal) evolves at the fastest rate, mirroring previous findings^22,62^, and it is the most morphologically diverse cranial region. Previous study has demonstrated that the suspensorium of anurans, comprising the quadratojugal, jaw joint, squamosal and pterygoid, is highly integrated, likely facilitating functional evolution of this region^79^ (and not only in anurans^80,81^). While evolutionary rate and disparity vary widely within and across cranial regions, both are high in the quadratojugal and adjacent surfaces (Fig. 4). Moreover, differences in evolutionary rates across microhabitats are pronounced for the jaw suspensorium cranial regions, further supporting that different feeding modes across microhabitats drive divergences in evolutionary tempo in the anuran skull (Fig. 5). There is extensive convergence in feeding mechanisms across Anura^82^, with feeding specialisations (hydrostatic elongation and suction feeding) occurring in some microhabitat specialists such as fossorial and aquatic taxa (although information on feeding mode and diet remains poor across frogs). Suction feeding, restricted to aquatic species, requires wide skulls^76,77^, resulting in a more lateralised jaw suspensorium. In contrast, hydrostatic elongation, which is observed in some fossorial frogs^83,84^ (and their close non-fossorial relatives, especially in the Microhylidae), requires minimal jaw opening, where only the lingual tip needs to leave the mouth^83^. This, and the small prey size (i.e. ants, termites) of some fossorial species^83–86^, allows the jaw suspensorium and the pectoral girdle to move anteriorly, which effectively eliminates the neck region^83,85^. Other fossorial species have wide, heavily ossified crania for consuming large prey including vertebrates (e.g. *Pyxicephalus adspersus*), and a recent study also identified higher rates of evolution, but not higher disparity, in hyperossified frogs, with a significant interaction with microhabitat^64^. Thus, the elevated rate of evolution and disparity across the crania of aquatic, fossorial and semi-fossorial frogs may be linked to the multiple feeding modes found within each of these microhabitats.

Hearing ability also may vary across microhabitats. The stapes displays the highest disparity in fossorial, semi-fossorial and aquatic taxa and show significant associations with adult microhabitat and size, but not larval feeding. Differences in anuran stapes evolution across microhabitats and its relatively high disparity may also be driven by the convergent losses, reappearances and re-losses of this structure^55^, which has also complicated its inclusion in most previous morphometric analyses. The stapes may be lost in frogs with little loss of acoustic communication^87,88^, and middle ear development can be heavily protracted^89^, suggesting the stapes may be ‘evolutionarily dispensable’. The other tympanic middle ear structures (tympanic membrane and tympanic annulus) are also repeatedly lost throughout anuran evolution^55^. Variation in extratympanic hearing sensitivity^87,90^ suggests species-specific extratympanic hearing mechanisms may exist^90^, for example sound detection through the mouth^88^. The different acoustic potentials of substrates^91^ may drive the evolution of alternative, extratympanic hearing mechanisms and promote lability in stapes morphological evolution across microhabitat specialists, meriting further investigation.

In addition to skull size and microhabitat, larval feeding mode is a significant albeit relatively weaker influence on anuran cranial evolution. The adult crania of frogs with and without a feeding larval stage do not differ notably in morphology (Supplementary Fig. 3). Of the factors studied here, there are the fewest significant associations between larval feeding and morphology of cranial regions. Previous study also demonstrates that taxa with or without actively feeding larvae do not differ in patterns of evolutionary integration for the cranium^79^, in contrast to the markedly different patterns observed among salamanders with different reproductive strategies^81^. Our results thus indicate that functional adaptations to the larval environment are less influential for adult morphology than are adaptations for the adult habitat and support previous suggestions for the independence of the larval and adult stages. These results further support previous studies identifying similarities in adult cranial morphology despite differences in early development in frogs and in marine invertebrates^37,92–94^. However, we found that taxa with non-feeding larvae consistently showed faster rates of evolution, significantly so for the majority of cranial regions, as well as higher disparity, suggesting that loss of a feeding larval stage still promotes morphological diversification in anurans.

As noted above, previous studies directly comparing larval and adult traits have variably found them to be correlated^45,95–97^ or independent^45,47,98,99^. These conflicting results among studies may reflect different effects across cranial elements, which were not treated individually in previous studies and which our results demonstrate display differential relationships with adult microhabitat and larval feeding. We found highly significant interactions between adult microhabitat and larval feeding for approximately half of the cranial elements (Supplementary Table 2). Elements with a weaker or no significant interaction between adult microhabitat and larval feeding, including the nasal, neopalatine, dorsal sphenethmoid and vomer, show large differences in evolutionary rate between taxa with or without a feeding larval stage. This difference was observed even when there is not a significant association between region shape and larval feeding. In contrast, those same elements show little difference in evolutionary rate across microhabitat categories, even if there is a significant association between shape and microhabitat. These results suggest two unexpected patterns. First, differences in shape and evolutionary rates are decoupled in many elements of the anuran cranium. This mosaicism in both the drivers of cranial shape and pace of evolution supports recent work demonstrating extensive modularity in the frog skull^79^. Second, there is a trade-off in the influence of adult microhabitat and larval feeding on evolutionary rates that varies across cranial elements. More specifically, differences in evolutionary rates among taxa with or without feeding larvae are most pronounced, and differences in evolutionary rates among microhabitats least pronounced, when there is not a significant interaction between adult microhabitat and larval feeding. In these cases, loss of an actively feeding larval stage consistently spurs significantly faster evolution of individual cranial regions.

It may be expected that frogs with non-feeding larvae have relaxed their constraints on certain aspects of larval cranial morphology, given the lack of selection pressures related to feeding at earlier stages of development. This may manifest as higher evolutionary rates, rather than a straightforward association with specific morphologies. Our finding that the mean evolutionary rate is higher for taxa with non-feeding larvae for nearly all cranial regions, and significantly so in most, provides strong support for the hypothesis of released constraints on cranial morphology. Direct development also has been hypothesised to promote morphological novelty across plethodontid salamanders^19,20^, suggesting that presence of a larval stage may constrain adult morphology. However, we would expect to observe this effect most strongly in the jaw suspensorium region, as frogs with feeding larvae exhibiting dramatic ontogenetic changes in diet, transitioning generally from small-mouthed microphagous suspension feeders to large-mouthed carnivorous adults^33,38–40^. The primary cranial shape change through metamorphosis results from a posterior translation of the quadrate bar (which supports the jaw joint)^40,100^, reflecting the change in feeding mode^100^. In direct developers (all of which have non-feeding larvae), the quadrate bar varies widely, forming in positions that correspond to larval, mid-metamorphic or adult stages^40^. A posterior translation of the quadratojugal (jaw joint) also contributes to the major axis of shape variation across adult frog crania (Fig. 2 and Supplementary Fig. 1), and the quadratojugal is the fastest-evolving cranial region in anurans. Yet we did not find a significant difference in evolutionary rate based on larval feeding for this structure or the adjacent and functionally related maxilla and squamosal. Morphological repatterning through ontogeny related to larval feeding mode may play a major role in adult morphological evolution. However, the wide range of ontogenetic trajectories among frogs and the retention of ancestral features in some taxa with non-feeding larvae^93^ complicates identification of a more subtle relationship between larval feeding and adult morphological evolution. Quantifying ontogenetic trajectories of cranial shape across the breadth of frog diversity—and even across different larval feeding modes and microhabitats—is a challenging task for future studies but would bring critical data towards refining our understanding of this relationship.

Overall, we interpret our results to indicate that adult microhabitat and body size shape large-scale differences in morphology for most structures, but both adult microhabitat and loss of actively feeding larvae promote diversification of cranial form. The relationship between larval feeding and adult morphology in anurans is likely to be more complex than that of size and microhabitat, with effects manifested within specific systems, whether clades, anatomical elements or microhabitats, but not at a broader phylogenetic scale. This effect may extend to specific morphologies, as well as evolutionary rate and disparity. For example, larval feeding has been suggested to influence adult feeding mode in aquatic amphibians, specifically with suction feeding adults arising predominantly from suction feeding larvae^97^. Our results similarly suggest that these effects of larval feeding mode are likely to manifest within specific structures and specific habitats rather than across the entire skull. Whether specific larval feeding modes and larval diets might differentially impact the disparity and rates of evolution of cranial structures remains unknown.

Another aspect of development that is more readily quantifiable is ossification sequence. Timing of bone formation varies across taxa, with, for example, earlier formation of jaw suspensorium bones in direct developers, related to their lack of larval feeding and earlier requirement of adult-mode feeding^28,101,102^. Thus, ossification sequence rank may reflect functional requirements that differentially constrain the morphological evolution of some cranial elements, as has been demonstrated in marsupial mammals^18^. We find strong evidence that the early onset of ossification constrains the evolution of individual skull elements, and that later-ossifying bones are more evolutionarily labile, with a strong positive relationship between median ossification sequence rank (relative timing) and both evolutionary rate and disparity (Fig. 6 and Supplementary Figs. 10,11). The early-ossifying parasphenoid, frontoparietal and occipital regions are involved in the protection of the brain and otic capsules^33,103^ and display the least restructuring during metamorphosis^28^, suggesting they experience strong functional constraints. In contrast, many later-ossifying bones, such as the vomer, are variably present across Anura (possibly a result of progenesis^43^ or miniaturisation^73^) and undergo greater morphological changes during metamorphosis^28^, suggesting their functions are less conserved and their morphologies thus less constrained. Previous studies have demonstrated that ossification in anurans occurs first in areas experiencing high stress^104^. Ossification sequence rank is thus possibly linked to functional demands^103^, with the later-ossifying bones being less functionally critical^28^ and thus less constrained in morphology. Interestingly, recent study has suggested that later-ossifying elements are also more evolutionarily integrated^79^, contrary to expectations that more functionally conservative regions may be more integrated. It may be that extensive variability in the late-ossifying elements, particularly with several elements that are variably present across anurans, requires tightly coordinated changes that drive high evolutionary integration within those elements, as well as greater modularity among elements. Overall, the results presented here support the long-standing hypothesis that early developmental stages are more evolutionarily conservative than late developmental stages^105,106^ and suggests that late ossification of cranial elements, and perhaps strong integration of those elements, may allow or even promote variability in form and function.

The coupling of dense phenotypic data with dense taxonomic sampling presented here has provided a framework with which a detailed, quantitative investigation into the drivers of phenotypic evolution across the anuran skull is achievable. Here we find microhabitat and size are the primary drivers of cranial morphological evolution across frogs, with aquatic and fossorial microhabitats associated with distinct cranial forms, faster evolutionary rates and greater morphological diversity. Evolutionary rate and disparity are particularly elevated in the jaw suspensorium regions, indicating that feeding mechanics related to microhabitat is likely a significant driver of anuran cranial evolution. Feeding mechanics also contributes to a ‘large size-wide skull’ pattern of allometry across anurans, with large prey specialists requiring wider skulls. In combination with similar results for caecilians^22,71^, we suggest that this pattern may form a general rule of ACREA. Surprisingly, taxa with and without feeding larvae do not notably differ in their cranial morphology. Although larval feeding mode is significantly associated with shape of most cranial elements, it has a much smaller effect size than microhabitat or size on cranial morphology. Significant differences in evolutionary rate and disparity are observed in half of the cranial elements examined between taxa with and without actively feeding larvae. Where differences do occur, they consistently support higher rates of evolution in taxa that have lost an actively feeding larval stage. Our results thus support both that larval feeding constrains at least the rate of morphological evolution and that amphibian larvae serve as a distinct developmental module^23^. Even if only a weak influence, functional pressures experienced earlier in ontogeny appear to still impact patterns of adult morphological evolution, and our results demonstrate a significant interaction between adult microhabitat and larval feeding across the skull and in most cranial regions. An interesting area for future research will be to conduct finer-scale comparisons of morphological evolution among anuran clades that have diversified into similar sets of microhabitat specialists but differ in their life histories. For example, do lineages with non-feeding larvae evolve more quickly to fill this morphospace than those with typical feeding tadpoles? Future research directly incorporating quantitative shape data through ontogeny and making explicit comparisons between clades with contrasting life histories or larval ecologies will be instrumental for further clarifying the complex interactions between adult and larval ecology and morphological evolution across >200 million years of frog evolution.

## Methods

### Specimens

We reconstructed and processed meshes from microCT scans of crania for 173 anuran species, including representatives from all extant frog families and the mummified Eocene *Thaumastosaurus gezei*^107^ (Fig. 2 and Supplementary Table 5). A recent phylogenetic analysis suggests *Thaumastosaurus gezei* is a ranoid with African affinities^107^, nested within the Natatanura clade from Frost et al.^108^. Although complete, undeformed 3D fossil material for anurans is limited, inclusion of any fossils increases our temporal sampling and has been demonstrated to improve estimates of evolutionary rate^109,110^. Meshes from microCT scans were created in Avizo Lite 9 (FEI Visualisation Sciences Group, Burlington, MA, USA) and VG Studio MAX^111^ and processed in Geomagic Wrap 2017 (3D Systems, Rock Hill, South Carolina, USA) to remove noise and small surface foramina, as previously described^112^. Scan information can be found in Supplementary Data 1.

### Phylogeny

We reconstructed the maximum clade credibility tree (MCC) from the posterior sample of the most recent, comprehensive, time-calibrated phylogeny of Anura^113^ using treeAnnotator. We pruned the MCC tree to match our species using the ‘drop.tip’ function in the R package ape v.5.3^114,115^ and modified it by adding four specimens without species assignments to their respective genera within the phylogeny. Specifically, *Raorchestes* sp., *Dendrobates* sp., *Capensibufo* sp. and *Xenorhina* sp. took the positions of *Raorchestes anili, Dendrobates auratus, Capensibufo rosei* and *Xenorhina varia*, respectively. In addition, the fossil specimen *Thaumastosaurus gezei* was added as sister taxon to the clade comprising *Ceratobatrachus*, *Aubria* and *Pyxicephalus*, as recovered from a recent phylogenetic analysis^107^. We further randomly drew 100 trees from the posterior distribution^113^ to assess the effect of phylogenetic uncertainties in downstream comparative analyses (the fossil taxon was excluded from the analyses of the 100 trees).

### Morphometric data collection

Fifteen cranial regions were defined (Fig. 2 and Supplementary Table 6) using 58 landmarks, 410 curve semilandmarks and 527 surface semilandmarks (Supplementary Tables 7–9). Landmarks and curve-semilandmarks were digitised in iDAV Landmark Editor v.3.6^116^, while surface semilandarks were applied to each cranium semi-automatically^112^ using the R package Morpho v.2.7^117^. Regions that were variably present across taxa were represented as ‘negligible regions’ when absent^22,112^ (Supplementary Table 5). The effect of negligible regions on trait integration was investigated previously for the dataset in the current study^79^, where the pattern of trait integration across the anuran skull was found to be near identical for datasets including and excluding specimens with negligible regions. Negligible regions represent the loss of that region and are therefore important to retain when accurately reconstructing evolutionary rate. Exclusion of negligible regions would underestimate evolutionary rate as the evolutionary loss of structures, an extreme form of variation and evolutionary change, would not be captured. Semilandmarks were slid to minimise bending energy globally, then all data were mirrored to improve Procrustes alignment^117^. Only right-side and midline data were retained for analysis to reduce redundant dimensionality^60–62,118^.

### Size

Log centroid size of the cranium was extracted from shape data during Procrustes superimposition and was used as a proxy of overall size (Supplementary Table 9).

### Habitat and larval feeding

We used functionally relevant, discrete categories for microhabitat and larval feeding, which were necessarily limited by data availability for most of the taxa in our dataset. Microhabitat was divided into the following seven categories: aquatic, semi-aquatic, arboreal, semi-arboreal, fossorial, semi-fossorial and terrestrial, based on adults observed outside of the breeding season. The habitat categorisation reflects the four main habitats identified across frogs^30^, with the recognition that intermediate forms between terrestrial and the other three habitats also exist^30^. In addition, these categories are in line with previous studies using habitat categories^31,32,119–121^. Ecological categories were based on predominant (or partial for ‘semi-’ categories) use of a substrate (water for aquatic, vegetation for arboreal and soil for fossorial) compared with land (terrestrial). Whilst fossorial species can dig head-first, forelimb first or hind limb first, and whilst some species found in burrows may not actively burrow, this information is not always available, and we do not have sufficient numbers of representatives from each category. We therefore grouped these into ‘fossorial’ (and ‘semi-fossorial’) categories, as this would capture general selection pressures associated with endogeicity (living in soil), as previously discussed for caecilians^22^. Furthermore, studies have recognised that whilst some frogs may burrow backwards, they move forward underground and excavate termite colonies by digging forwards through tunnels e.g. *Rhinophrynus dorsalis*^83^, so would likely experience similar selection pressures underground to forward fossorials. For a detailed investigation into burrowing influences on morphological evolution for myobatrachid frogs, we refer to Vidal-García and Keogh^3^. Data from the primary literature were used, as well as categories established in previous studies^31,32^ (Supplementary Data 1). Three taxa did not have microhabitat information available and were excluded from analyses of microhabitat (Habitat: *N* = 170).

Larval feeding categorisation used the following definitions^38^: taxa with feeding (exotrophic) larvae (i.e. feed on external food sources) and taxa with non-feeding (endotrophic) larvae (i.e. solely provisioned with yolk). The larval feeding categorisation distinguishes frogs experiencing extrinsic selection pressures from two environments (larval and adult), to those experiencing only pressures from the adult environment. Whilst direct developers can vary in their ontogeny, all are assumed to be endotrophic^122^. Trophic egg feeding or feeding on skin secretions were both counted as ‘feeding’ larval forms. We use this categorisation rather than metamorphic vs direct developing because metamorphic forms include those with and without feeding larvae and thus that categorisation does not capture the differences in cranial function and selection pressures imposed by a feeding larval stage. Data were taken from the primary literature (Supplementary Data 1). Ten taxa did not have information on larval feeding and were excluded from those analyses (larval feeding *N* = 163; 162 taxa have data on both larval feeding and microhabitat). While finer categories of both developmental and ecological traits are possible, these broader categorisations of adult microhabitat and larval feeding are necessary for robust statistical analyses, which require a minimum of five species per group. Other traits would suffer from much more missing data, while finer categories would result in many bins with only few species within them.

### Ossification timing

Data on relative timing of cranial bone ossification were taken from Weisbecker and Mitgutsch (2010)^43^ using median rank position for all bones except the stapes (which is not included in that study). The occipital region defined in this study corresponds to the oto-occipital bone, and the otic region corresponds to the prootic bone (Supplementary Table 5).

### Data analyses

#### Cranial shape

Shape analyses were conducted using the R package geomorph v3.1.3^123^ unless otherwise noted. PCs analysis was used to assess the main axes of shape variation for the whole cranium and individually for the 15 cranial regions. Phylomorphospaces were plotted in phytools v0.6-60^124^, and the primary axes of shape variation were visualised by generating extreme shapes along the main PC axes. Size-related shape variation (allometry) was extracted for the whole cranium using the ‘procD.lm’ function, and we repeated the morphospace analyses on the residuals of shape variables regressed against log centroid size to visualise the distribution of microhabitat and larval feeding categories independent of allometry. Morphologies representing the extreme shapes along each PC axis that represented over 5% of the variation were visualised for the entire cranium, and along PC1 for each cranial region. For region-specific analyses of shape, only specimens with the relevant region present were retained. It is important to note that the directionality of the PC axes was arbitrary (Supplementary Fig. 2), as the PCAs were conducted on individual cranial regions.

Type II phylogenetic MANOVAs (phylogenetic regressions) were performed on the landmark and semilandmark data for the whole cranium and individual cranial regions with log centroid size, microhabitat and larval feeding as predictors using the ‘mvgls' and ‘manova.gls' functions in the R package mvMORPH 1.1.4^125^. Specifically, we fitted multivariate phylogenetic linear models with Pagel’s lambda by penalised likelihood using ‘mvgls'^126^ and assessed the significance of each predictors in this model by type II MANOVA tests using the ‘manova.gls' function with Pillai’s statistic and 1000 permutations^127^. Using Pagel’s lambda corresponds to fitting a phylogenetic mixed model which allows accounting for departure from Brownian motion and usually provides increased flexibility in estimating the error structure^127–130^. MANOVAs were performed on both the MCC tree and from the 100 trees randomly sampled in the posterior distribution^113^ to assess the effect of phylogenetic uncertainties.

#### Ancestral shape and trait estimation

Ancestral cranial morphology for anurans was estimated by maximum likelihood using the ‘anc.recon’ function in the R package Rphylopars v0.2.11^131–133^, and the ancestral states for microhabitat and larval feeding were estimated using the ‘ace’ function in *ape* v5.3.

#### Disparity and evolutionary rate

Evolutionary rate and morphological disparity (measured as Procrustes variance scaled by number of landmark/semilandmarks) were calculated for each cranial region across the entire dataset and for each microhabitat and larval feeding category after removing the effect of size (log cranial centroid size), using the state-specific Brownian motion (BMM) model in the ‘mvgls’ function in mvMORPH and ‘morphol.disparity’ functions in geomorph. The reconstructed history for microhabitat and larval feeding categories on which BMM models were fitted was obtained through stochastic character mapping across the sample of 100 trees using the ‘make.simmap’ function and an ‘ARD’ model in the R package phytools. Model fit in ‘mvgls’ was performed by jointly estimating the contribution of measurement error and intraspecific variation using the option ‘error=TRUE' to mitigate the potential sources of BM departure that may lead to evolutionary rates inflation. Overall morphological disparity and evolutionary rate—taken as the average of rates estimated across coordinates and calculated across a sample of 100 trees—for each region were compared to ossification sequence rank (except for the stapes, which lacks data on ossification timing) using Spearman’s rank correlation analysis. Disparity and evolutionary rate were also calculated for each individual landmark and semilandmark in geomorph to visualise their distributions across the anuran skull on a finer scale.

### Reporting summary

Further information on research design is available in the Nature Research Reporting Summary linked to this article.

## Supplementary information

Supplementary Information

Description of Additional Supplementary Files

Supplementary Data 1

Reporting Summary

## Data Availability

Meshes are available on the online repositories Morphosource.org and phenome10k.org for use by other researchers. Morphosource DOIs are detailed in Supplementary Data 1. Landmark and semilandmark data are available at https://github.com/anjgoswami/frogs_modularity^134^. All other trait data and sources (e.g. ecological and developmental traits) are provided in Supplementary Data 1.
