# Supplementary material for: Size, microhabitat, and loss of larval feeding drive cranial diversification in frogs
Source: Nat Commun. 2021 May 4;12:2503. doi: 10.1038/s41467-021-22792-y (PMC8096824; doi:10.1038/s41467-021-22792-y)
Supplement: Supplementary file 1 — Supplementary Information [file 41467_2021_22792_MOESM1_ESM.pdf]

Supplementary Information

SIZE, MICROHABITAT, AND LOSS OF LARVAL FEEDING DRIVE CRANIAL DIVERSIFICATION IN FROGS

This document includes the following supplementary contents:

**Supplementary Note 1.** Additional results details	2
**Supplementary Figure 1.** Cranial morphological changes along PC1, PC2, PC3, and PC4.	4
**Supplementary Figure 2.** Shape variation for each cranial region defined in this study.	5
**Supplementary Figure 3.** Phylomorphospace of all 173 anuran specimens, indicating ranges of each life history strategy on PC1 and PC2.	6
**Supplementary Figure 4.** Range of cranial sizes for specimens from each habitat	7
**Supplementary Figure 5.** Distribution of life history strategies for each habitat.	7
**Supplementary Figure 6.** Phylomorphospace of all 173 anuran specimens, indicating ranges of each habitat on PC1 and PC2.	8
**Supplementary Figure 7.** Cranial morphological changes along PC1, PC2, PC3, and PC4 for data that are size-corrected.	9
**Supplementary Figure 8.** Per-region disparity, corrected for cranial region semi/landmark number, for each of the seven habitat categories	10
**Supplementary Figure 9.** Per-region disparity, corrected by semi/landmark number, and evolutionary rate for the two life history categories.	11
**Supplementary Figure 10.** Relationship between ossification sequence rank and disparity for each cranial region.	12
**Supplementary Figure 11.** Ossification sequence rank and evolutionary rate without error	12
**Supplementary Table 1.** Summary of the PC axes for the full landmark and semilandmark dataset.	13
**Supplementary Table 2.** pMANOVA results with interactions and distribution of 100 phylogenetic trees	17
**Supplementary Table 3.** Pairwise significance values for differences in cranial disparity for the seven habitats, for each cranial region	19
**Supplementary Table 4.** Results for differences in cranial disparity and rate of cranial evolution between taxa with or without feeding larvae, for each cranial region	23
**Supplementary Table 5.** Definition of cranial regions	24
**Supplementary Table 6.** Landmark definitions used in this study	27
**Supplementary Table 7.** Curve definitions used in this study	30
**Supplementary Table 8.** Number of surface points within each cranial region	33
**Supplementary Table 9.** Centroid size ('Csize') for each specimen	34
**References for Supplementary Information**	36

Supplementary Note 1

Results

Cranial morphology

The third principal component (PC) axis accounted for 8% of shape variation, with the main shape changes (from negative to positive) including the reduction of the maxillary arcade and the expansion of the pterygoid (Supplementary Fig. 1). The fourth PC axis accounted for 5% of shape variation, with the negative extreme associated with wider premaxillary and maxillary palatal shelves and a relatively larger pterygoid (Supplementary Fig. 1).

Individual cranial regions exhibited considerable morphological variation along PC1 (Supplementary Fig. 2). The maxilla region variably contributed to the maxillary arcade; the maxilla could extend posteriorly and articulate with the quadratojugal, completing the maxillary arcade (PC1 min, *Hypsiboas boans*), or the maxilla and quadratojugal did not articulate, leaving an incomplete maxillary arcade (PC1 max, *Balebreviceps hillmani*). The frontoparietal region varied from being laterally extended, dorsoventrally tall, and domed in shape in lateral view (PC1 max, *Hemisus guineensis*), to being mediolaterally compressed and flat in lateral view (PC1 min, *Barbourula busuangensis*). The parasphenoid exhibited the main variation in the presence and size of the lateral processes; the parasphenoid could be triradiate in shape, with lateral wings larger than the anterior process (PC1 min, *Peltophryne guentheri*), or the lateral processes could be absent (PC1 max, *Xenopus laevis*). The main variation exhibited along PC1 for the squamosal was the size of the rami; the anterior and posterior rami could be large (PC1 min, *Barbourula busuangensis*), or more reduced in size (PC1 max *Nasikabatrachus sahyadrensis*). The pterygoid varied in the size of the medial ramus; the medial ramus could be thin (PC1 max, *Myobatrachus gouldii*) or expanded anteroposteriorly (PC1 min, *Pipa parva*). The nasal could extend far ventrally, contacting the maxilla (PC1 max, *Ceratophrys aurita*) or the nasal could be reduced in size and isolated from other ossified material (PC1 min, *Rheobatrachus silus*). The occipital region could be mediolaterally expanded (PC1 min, *Hemiphractus proboscideus*) or mediolaterally compressed (PC1 max, *Pipa parva*), and the otic region varied from having a concave surface (PC1 max, *Ceratobatrachus guentheri*) to a convex surface (PC1 min, *Brachycephalus ephippium*). Along PC1, the quadratojugal varied from possessing an elongate maxillary process and wide articular surface (PC1 min, *Conraua beccarii*), to possessing a shorter, taller maxillary process, and a less ossified articular surface (PC1 max, *Nasikabatrachus sahyadrensis*). The vomer region varied from being anteroposteriorly elongate (PC1 max, *Xenorhina* sp.), to anteroposteriorly compressed, with the anterior process positioned more laterally (PC1 min, *Ameerega parvula*). The sphenethmoid (dorsal) region varied in the extent of its anterior projection, from projecting anteriorly in between the paired nasals (PC1 max, *Anhydrophryne rattrayi*) to remaining posterior to the nasals (PC1 min, *Hemiphractus proboscideus*). The sphenethmoid (ventral) region also varied in its anterior projection, from being present only lateral to the parasphenoid (PC1 min, *Myobatrachus gouldii*) to projecting anteriorly to the parasphenoid (PC1 max, *Arthroleptella lightfooti*). For the remaining cranial regions, the orientation (relative to the cranium) was a major aspect of the variation along PC1. The stapes varied from a mediolateral orientation (PC1 max, *Thaumastosaurus gezei*) to the lateral tip of the stapes pointing anteroventrally (PC1 min, *Pipa pipa* and *Myobatrachus gouldii*). However, the stapes of the mummified *Thaumastosaurus gezei* is 'almost in functional position'[1], so its extreme morphology may be partially an artefact of preservation. The premaxilla region exhibited the main variation in the orientation of the alary process, from the tip orienting anterodorsally (PC1 min, *Balebreviceps hillmani*) to dorsally (PC1 max, *Pristimantis chiastonotus*). Finally, the neopalatine was reduced in width with an anterolaterally oriented tip at one extreme of PC1 (PC1 min, *Hypsiboas boans*), and relatively wider with a laterally oriented tip at the other extreme (PC1 max, *Stumpffia pygmaea*).

Evolutionary rates and disparity

The crania of fossorial, semi-fossorial, and aquatic species all exhibited significantly higher disparity (whole skull Procrustes variance = 0.018 for aquatic and semi-fossorial and 0.019 for fossorial) than the crania of terrestrial (0.008), arboreal (0.009), semi-arboreal (0.007) and semi-aquatic (0.007)

species (pairwise significances in Supplementary Table 3; all disparity results for larval feeding mode in Supplementary Table 4).

Supplementary Figures

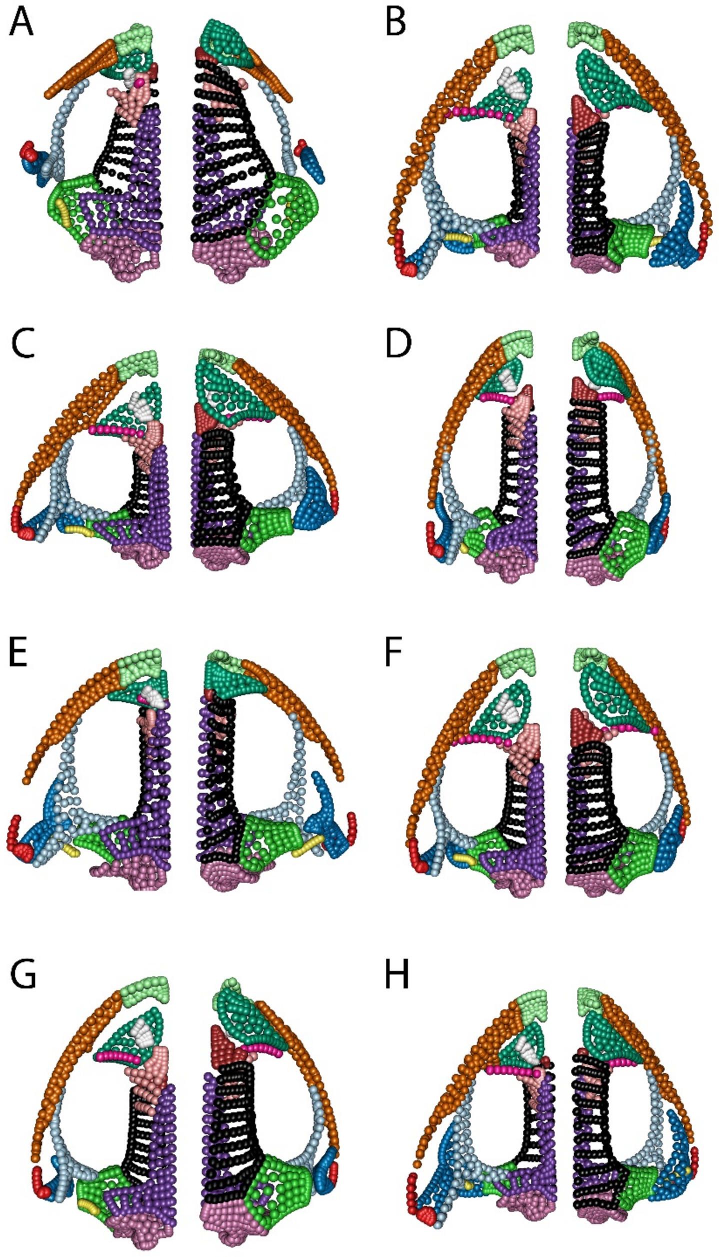

Supplementary Figure 1. Cranial morphological changes along PC1, PC2, PC3, and PC4. Reconstructed morphologies represented by positive (A) and negative (B) ends of PC1, positive (C) and negative (D) ends of PC2, positive (E) and negative (F) ends of PC3, and positive (G) and negative (H) ends of PC4, all shown in ventral (left) and dorsal (right) aspects. Each reconstruction is coloured by cranial region, as follows: Occ (light purple): occipital; FP (black): frontoparietal; Qj (red): quadratojugal; Max (orange): maxilla; Na (green): nasal; Neo (hot pink): Neopalatine; Otic (lime green): prootic; Pm (pale green): premaxilla; PS (purple): parasphenoid; Pt (light blue): pterygoid; SphD (brown): sphenethmoid (dorsal); SphV (light pink): sphenethmoid (ventral); Sq (blue): squamosal; St (yellow): stapes; Vo (grey): vomer. PC axes accounted for 28%, 14%, 8% and 5% of total cranial morphological variation, respectively.

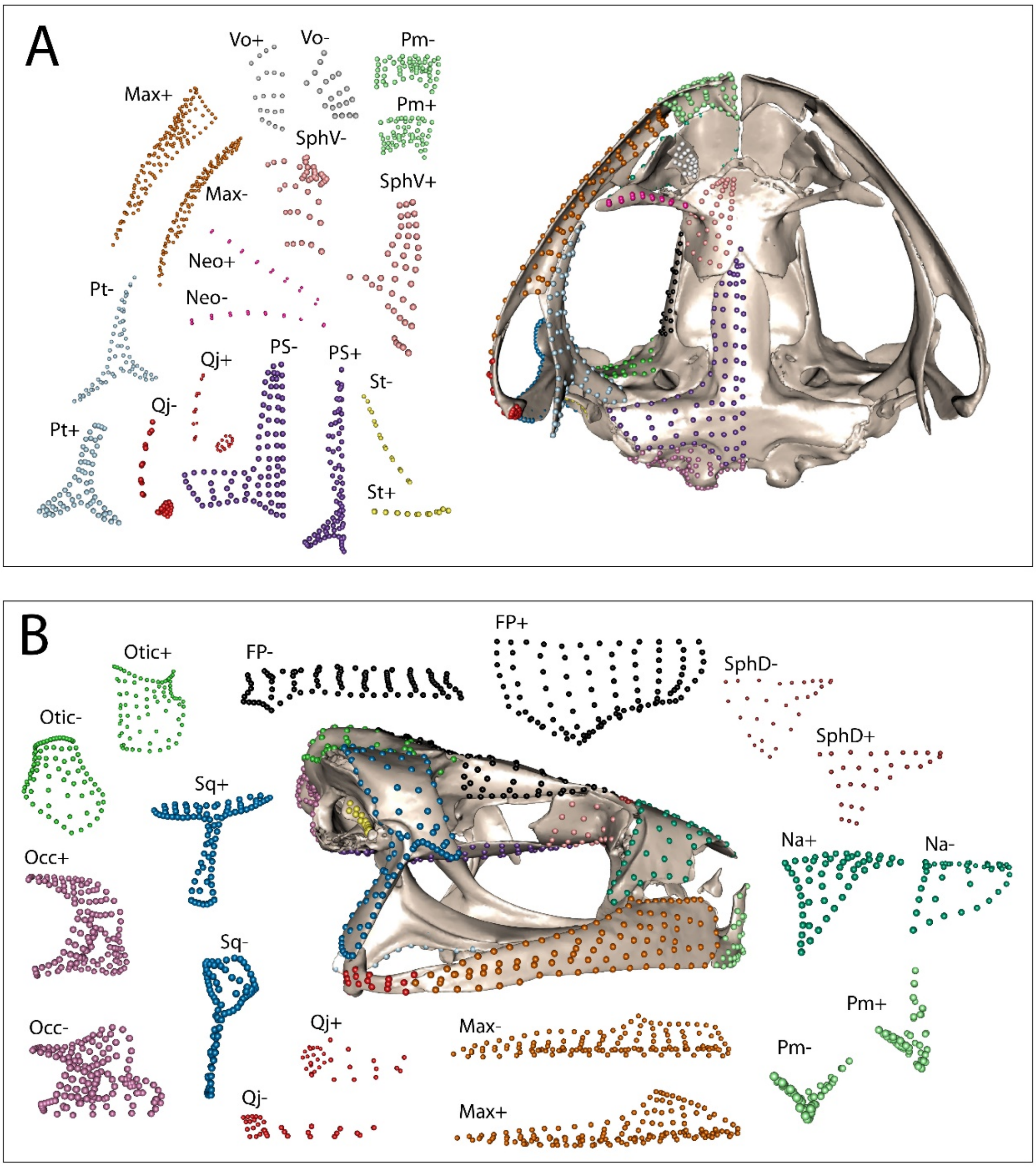

Supplementary Figure 2. Shape variation for each cranial region defined in this study. The landmark and semilandmark data are coloured by cranial region and displayed on *Adenomus kelaartii* (FMNH: Amphibians and Reptiles: 1580) in (A) ventral and (B) lateral aspects. The positive (+) and negative (-) shape extremes for each cranial region are displayed in (A) ventral and (B) lateral aspects, with the exception of the frontoparietal (FP), sphenethmoid (dorsal) (SphD) and otic (Otic) regions (dorsal aspect) and the occipital (Occ) region (posterior aspect). All regions are displayed in one view, except the maxilla, premaxilla and quadratojugal, which are displayed in both lateral and ventral views. Extreme shapes were generated from individual region PCAs and directionality of axes is arbitrary, so the PC axes do not necessarily align, and the extreme shapes are not to scale. Specimens with an absent region were removed from the PCAs for the relevant cranial regions, for visualisation purposes only. Regions are as follows: Occ (light purple): occipital; FP (black): frontoparietal; Qj (red): quadratojugal; Max (orange): maxilla; Na (green): nasal; Neo (hot pink): Neopalatine; Otic (lime green): prootic; Pm (pale green): premaxilla; PS (purple): parasphenoid; Pt (light blue): pterygoid; SphD (brown): sphenethmoid (dorsal surface); SphV (light pink): sphenethmoid (ventral surface); Sq (blue): squamosal; St (yellow): stapes; Vo (grey): vomer.

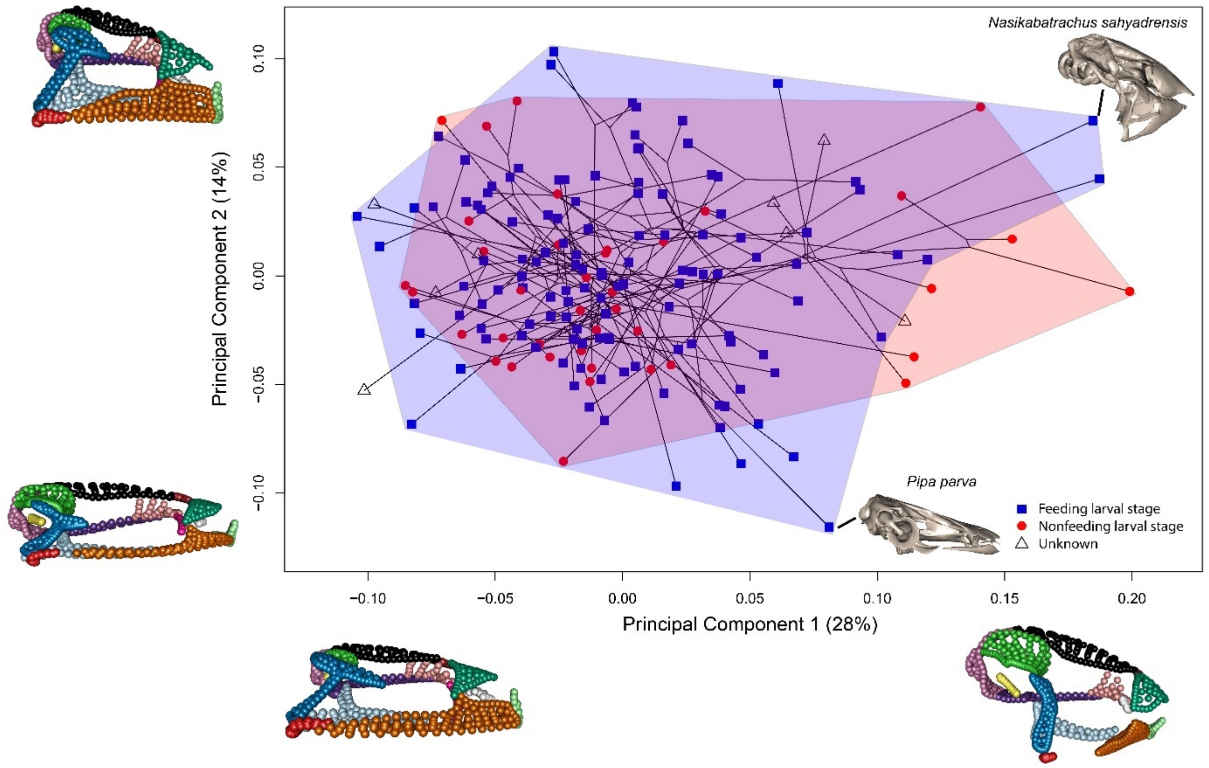

Supplementary Figure 3. Phylomorphospace of all 173 anuran specimens, indicating ranges of each life history strategy on PC1 and PC2. Symbols represent presence or absence of a feeding larval stage. Shaded regions illustrate morphospace occupation for taxa with feeding (blue) and non-feeding (red) larvae. Two specimen meshes are included to demonstrate the general shape differences between fossorial (top, *Nasikabatrachus sahyadrensis*, CESF:203) and aquatic (bottom, *Pipa parva*, UF: Herp:37924) species. Extreme shapes along PC1 and PC2 are visualised by deforming the shape data along each axis.

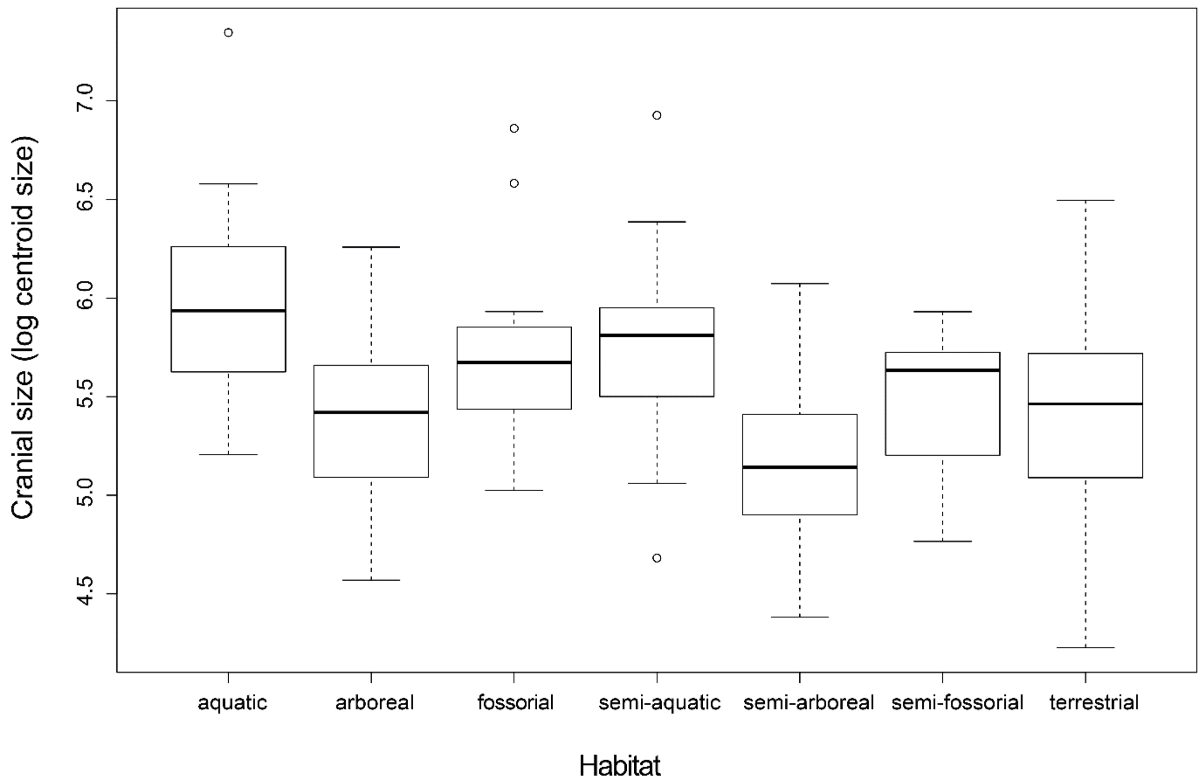

Supplementary Figure 4. Range of cranial sizes for specimens from each habitat. Sample sizes for each category are: aquatic (13), arboreal (39), fossorial (16), semi-aquatic (21), semi-arboreal (10), semi-fossorial (8), terrestrial (63). Box bounds represent the median with the first and third quartile, while whiskers indicate the minimum and maximum values within 1.5x the interquartile range.

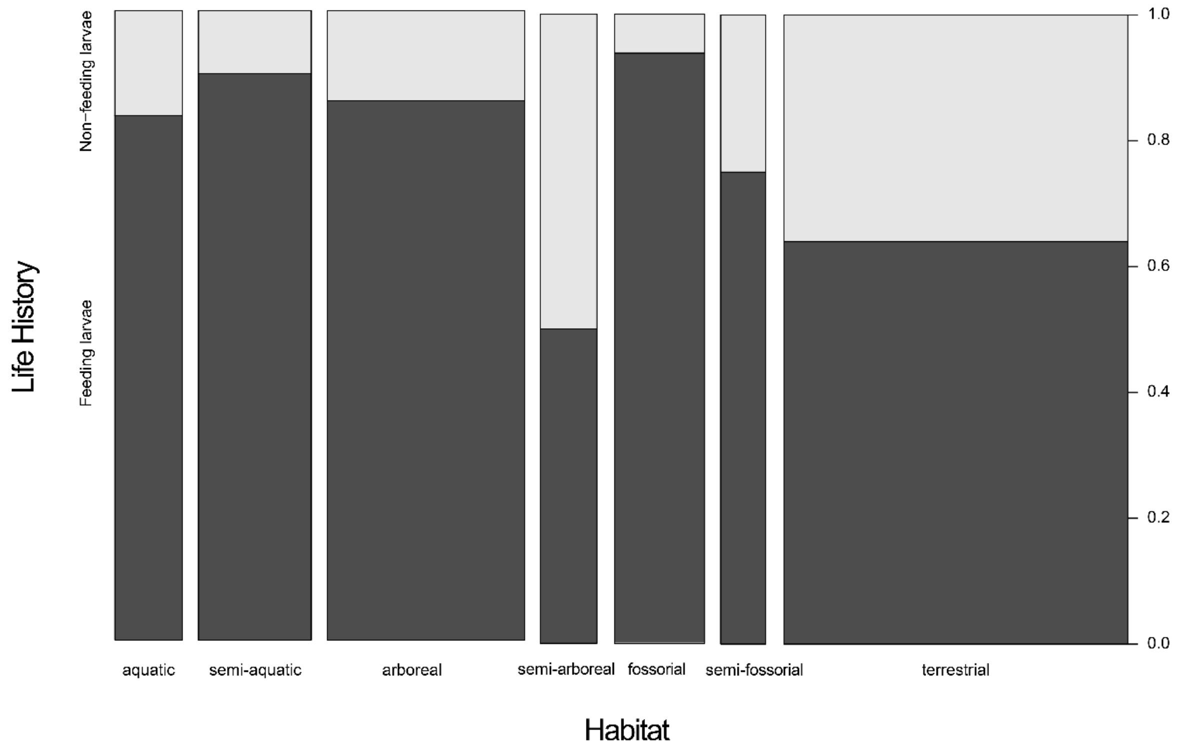

Supplementary Figure 5. Distribution of life history strategies for each habitat.

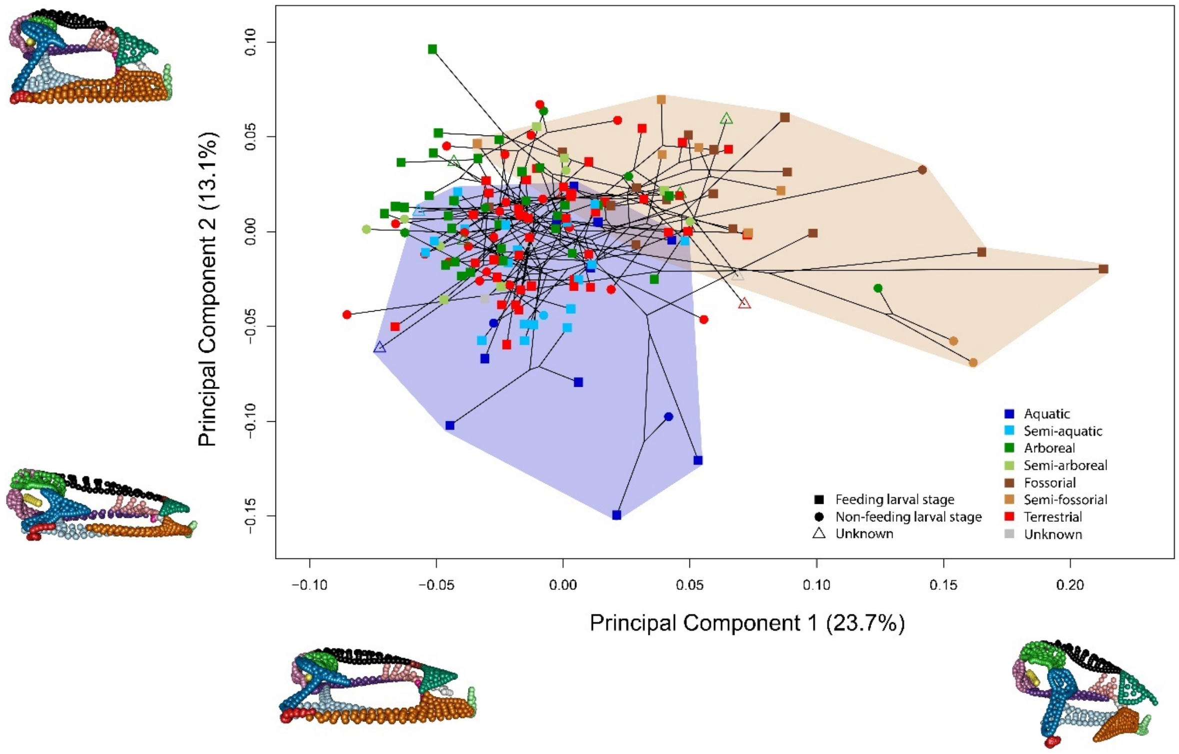

Supplementary Figure 6. Phylomorphospace of all 173 anuran specimens, indicating ranges of each habitat on PC1 and PC2. Symbols represent presence or absence of a feeding larval stage. Shaded regions illustrate morphospace occupation for taxa with fossorial/semi-fossorial (brown) and aquatic/semi-aquatic (blue) habitats. Extreme shapes along PC1 and PC2 are visualised by deforming the shape data along each axis. Data are size-corrected.

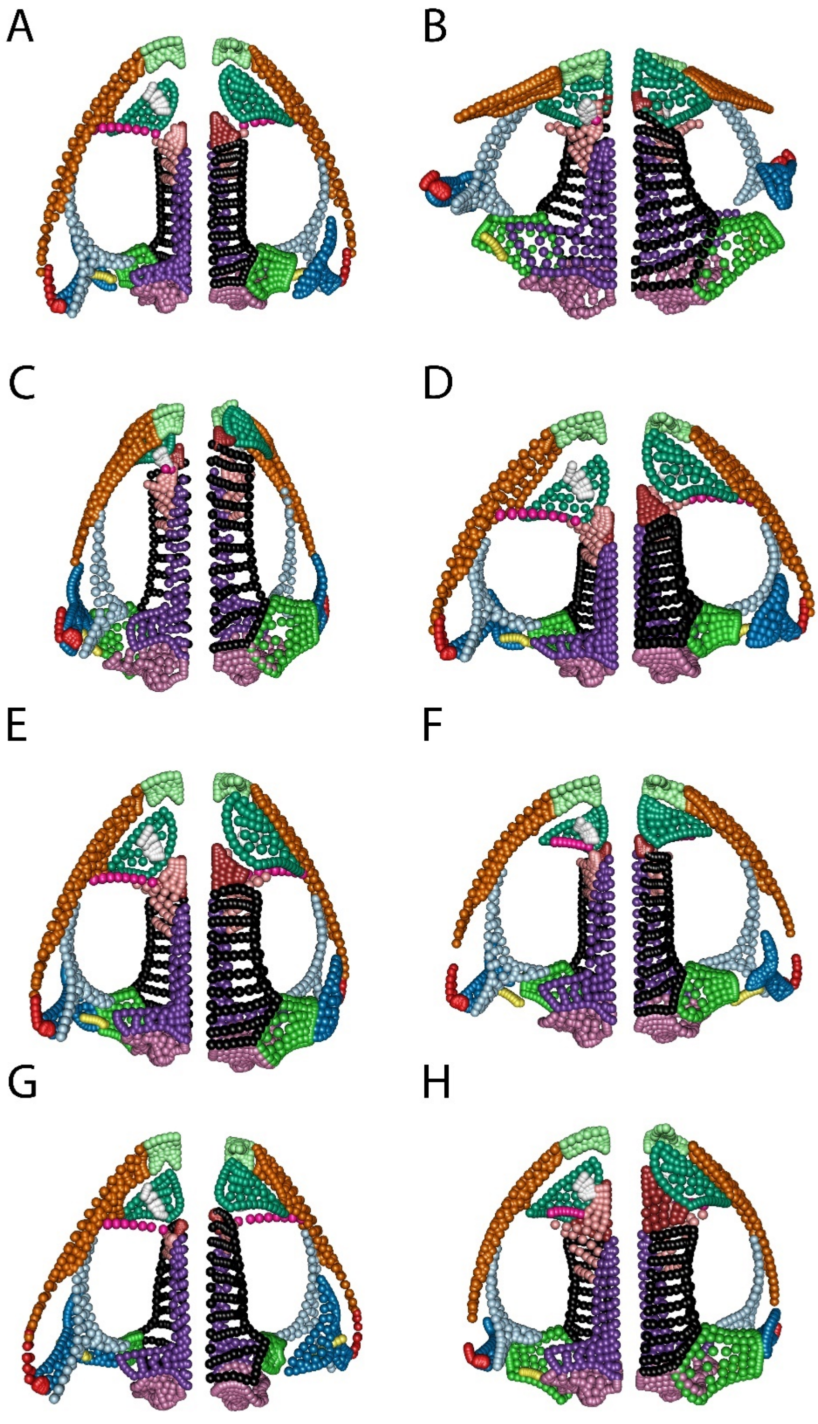

Supplementary Figure 7. Cranial morphological changes along PC1, PC2, PC3, and PC4 for data that are size-corrected. Reconstructed morphologies represented by positive (A) and negative (B) ends of PC1, positive (C) and negative (D) ends of PC2, positive (E) and negative (F) ends of PC3, and positive (G) and negative (H) ends of PC4, all shown in ventral (left) and dorsal (right) aspects. Each reconstruction is coloured by cranial region, as follows: Occ (light purple): occipital; FP (black): frontoparietal; Qj (red): quadratojugal; Max (orange): maxilla; Na (green): nasal; Neo (hot pink): Neopalatine; Otic (lime green): prootic; Pm (pale green): premaxilla; PS (purple): parasphenoid; Pt (light blue): pterygoid; SphD (brown): sphenethmoid (dorsal); SphV (light pink): sphenethmoid (ventral); Sq (blue): squamosal; St (yellow): stapes; Vo (grey): vomer.

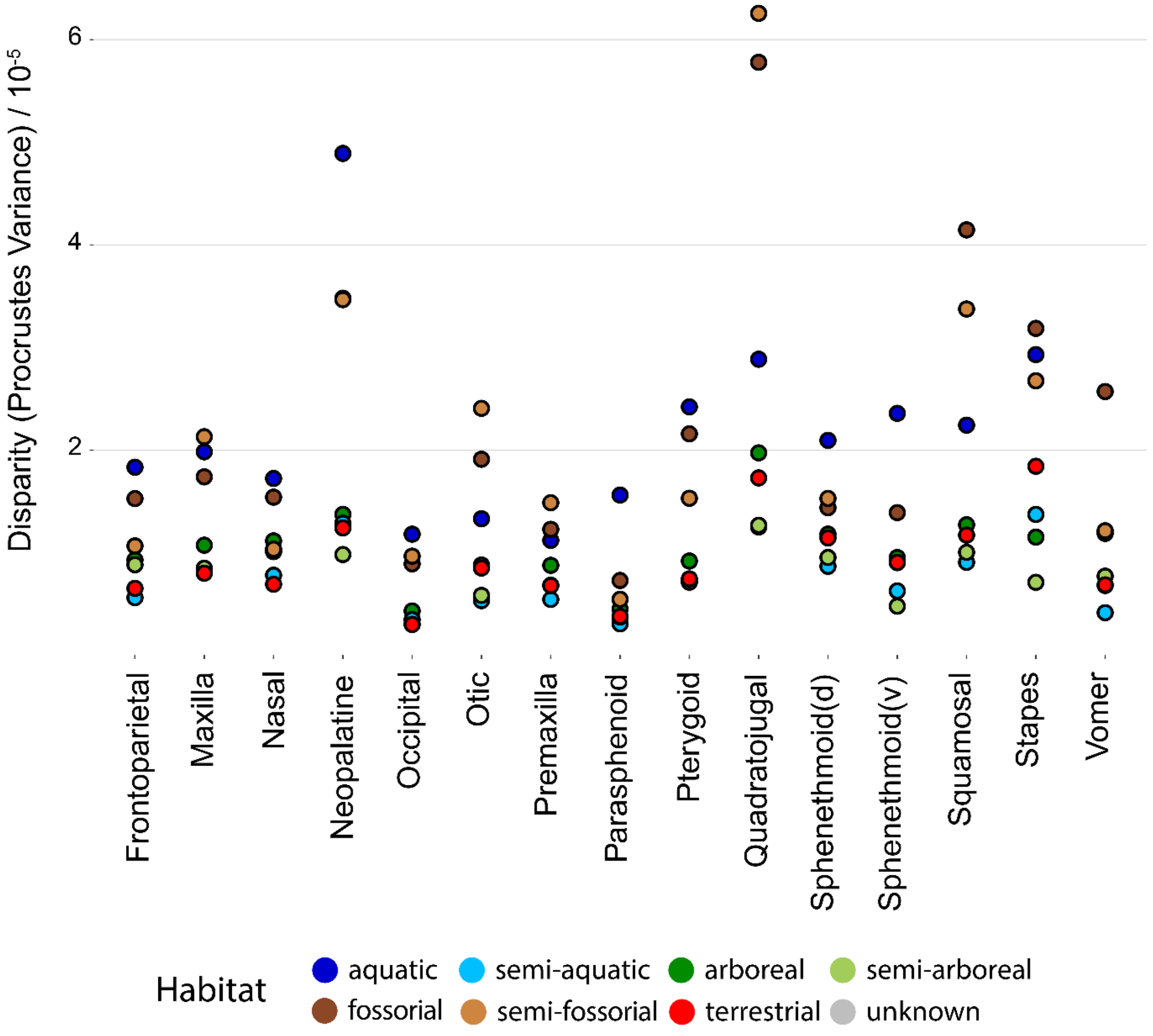

Supplementary Figure 8. Per-region disparity, corrected for cranial region semi/landmark number, for each of the seven habitat categories. Data are size-corrected.

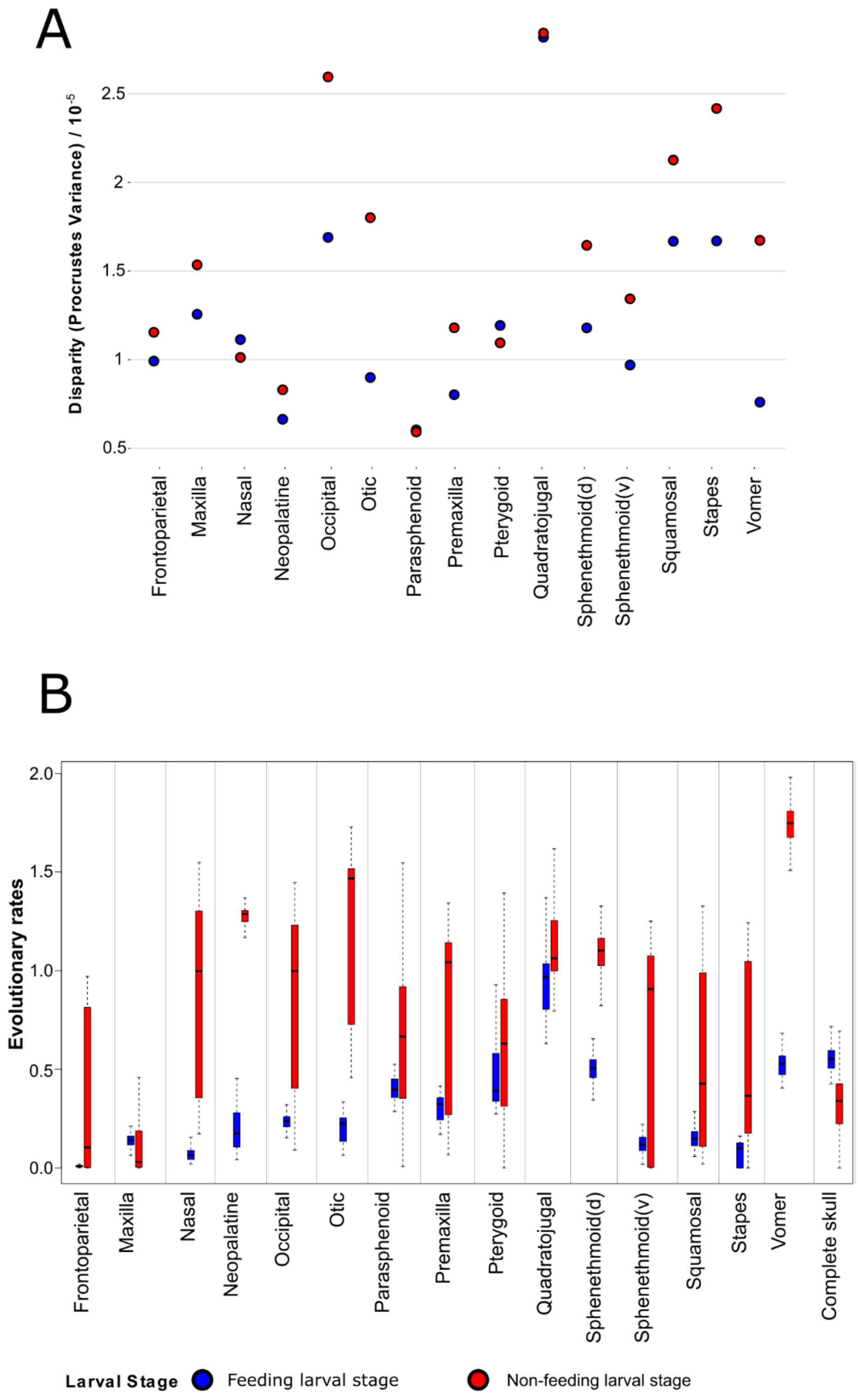

Supplementary Figure 9. Per-region disparity, corrected by semi/landmark number (A) and evolutionary rate (B) for taxa with (N = 124) and without (N = 39) larval feeding. Ranges for B are based on a random sample of 100 phylogenetic trees. Box bounds represent the median with the first and third quartile, while whiskers indicate the minimum and maximum values within 1.5x the interquartile range. Intraspecific variation and measurement error were jointly estimated along with evolutionary rates during model fit to account for uncertainty in traits values that may cause model departures.

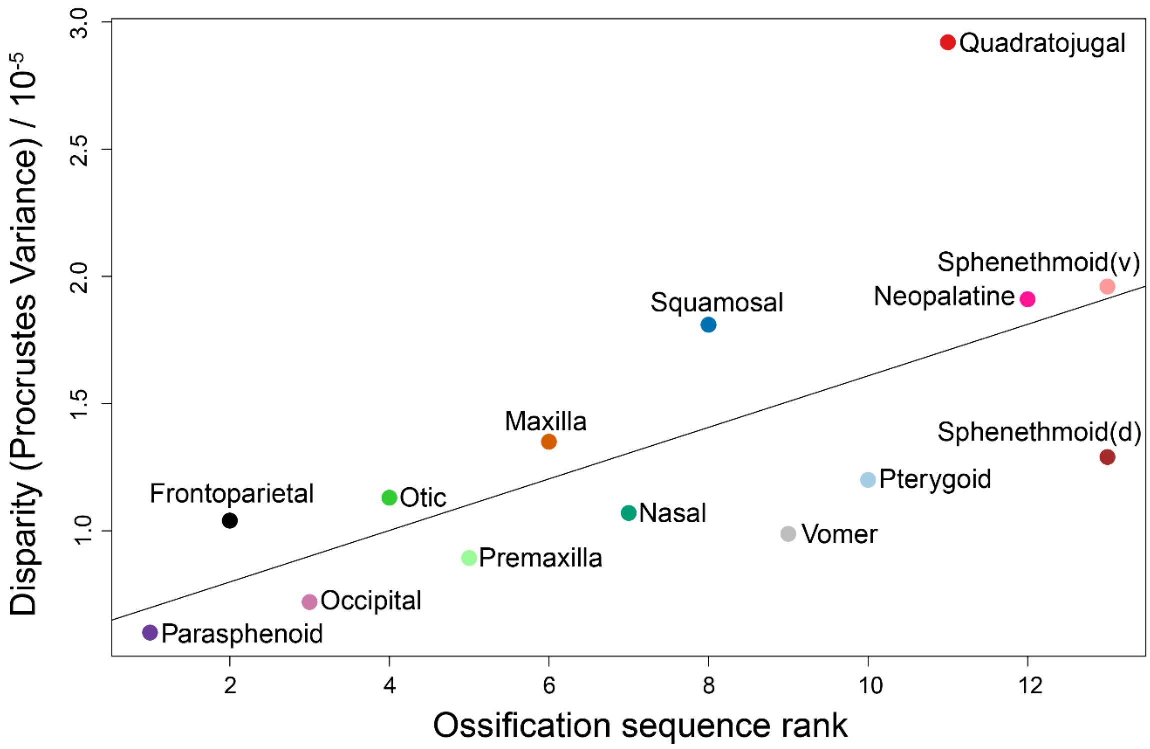

Supplementary Figure 10. Relationship between ossification sequence rank and disparity for each cranial region. Spearman's rank correlation of evolutionary rate with ossification sequence rank was significant (two-sided, Spearman's rho = 0.76, p = 0.0015).

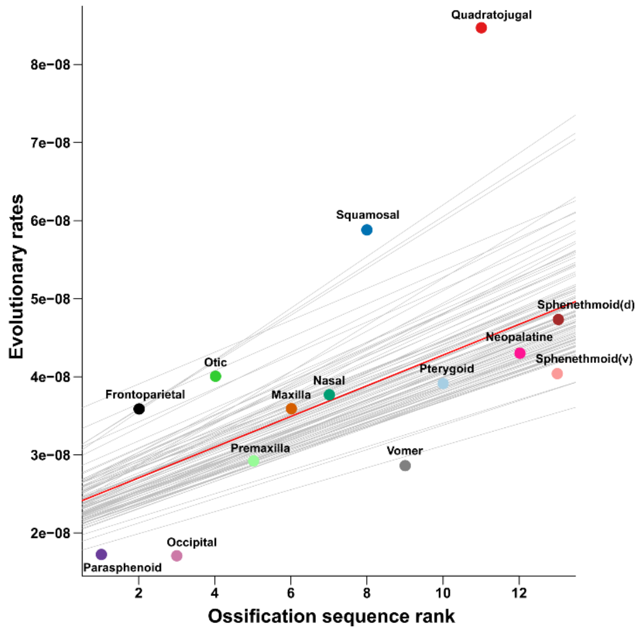

Supplementary Figure 11. Ossification sequence and evolutionary rate. Ossification sequence rank versus evolutionary rate for each cranial region, with evolutionary rates without error. All tests are one-sided, with significances based on permutations (n = 999). Two-sided Spearman's rank correlation of evolutionary rate with ossification sequence rank was significant (mean Spearman's rho = 0.61, $p < 0.01$).

Supplementary Tables

Supplementary Table 1. Summary of the PC axes for the full landmark and semilandmark dataset. 46 of the 172 PC axes were required to explain 95% of cranial shape variation.

	**PC1**	**PC2**	**PC3**	**PC4**	**PC5**
Standard deviation	0.057274	0.040853	0.030468	0.024258	0.022726
Proportion of Variance	0.2795	0.1422	0.0791	0.05014	0.044
Cumulative Proportion	0.2795	0.4217	0.50079	0.55093	0.59494
	**PC6**	**PC7**	**PC8**	**PC9**	**PC10**
Standard deviation	0.020868	0.019555	0.017219	0.016978	0.016295
Proportion of Variance	0.0371	0.03258	0.02526	0.02456	0.02263
Cumulative Proportion	0.63204	0.66462	0.68988	0.71444	0.73707
	**PC11**	**PC12**	**PC13**	**PC14**	**PC15**
Standard deviation	0.014362	0.013588	0.013248	0.012549	0.011563
Proportion of Variance	0.01757	0.01573	0.01495	0.01342	0.01139
Cumulative Proportion	0.75464	0.77037	0.78533	0.79874	0.81014
	**PC16**	**PC17**	**PC18**	**PC19**	**PC20**
Standard deviation	0.011173	0.010621	0.010396	0.010165	0.009781
Proportion of Variance	0.01064	0.00961	0.00921	0.0088	0.00815
Cumulative Proportion	0.82077	0.83038	0.83959	0.8484	0.85655
	**PC21**	**PC22**	**PC23**	**PC24**	**PC25**
Standard deviation	0.009348	0.008902	0.008557	0.008326	0.007968
Proportion of Variance	0.00745	0.00675	0.00624	0.00591	0.00541
Cumulative Proportion	0.86399	0.87074	0.87698	0.88289	0.8883
	**PC26**	**PC27**	**PC28**	**PC29**	**PC30**
Standard deviation	0.007853	0.007564	0.007383	0.007179	0.006862
Proportion of Variance	0.00525	0.00488	0.00464	0.00439	0.00401
Cumulative Proportion	0.89356	0.89843	0.90307	0.90747	0.91148
	**PC31**	**PC32**	**PC33**	**PC34**	**PC35**
Standard deviation	0.006568	0.006222	0.005981	0.005891	0.005726
Proportion of Variance	0.00368	0.0033	0.00305	0.00296	0.00279
Cumulative Proportion	0.91515	0.91845	0.9215	0.92446	0.92725
	**PC36**	**PC37**	**PC38**	**PC39**	**PC40**
Standard deviation	0.005619	0.005445	0.005408	0.005237	0.005092
Proportion of Variance	0.00269	0.00253	0.00249	0.00234	0.00221
Cumulative Proportion	0.92994	0.93247	0.93496	0.9373	0.93951
	**PC41**	**PC42**	**PC43**	**PC44**	**PC45**
Standard deviation	0.00504	0.004889	0.004801	0.004755	0.004656
Proportion of Variance	0.00216	0.00204	0.00196	0.00193	0.00185
Cumulative Proportion	0.94167	0.94371	0.94567	0.9476	0.94944
	**PC46**	**PC47**	**PC48**	**PC49**	**PC50**
Standard deviation	0.004591	0.004427	0.004353	0.00427	0.004188
Proportion of Variance	0.0018	0.00167	0.00161	0.00155	0.00149
Cumulative Proportion	0.95124	0.95291	0.95452	0.95608	0.95757
	**PC51**	**PC52**	**PC53**	**PC54**	**PC55**
Standard deviation	0.004043	0.003942	0.003889	0.003784	0.003761
Proportion of Variance	0.00139	0.00132	0.00129	0.00122	0.00121

Cumulative Proportion	0.95897	0.96029	0.96158	0.9628	0.964
	**PC56**	**PC57**	**PC58**	**PC59**	**PC60**
Standard deviation	0.003684	0.003615	0.003523	0.00347	0.003442
Proportion of Variance	0.00116	0.00111	0.00106	0.00103	0.00101
Cumulative Proportion	0.96516	0.96627	0.96733	0.96836	0.96937
	**PC61**	**PC62**	**PC63**	**PC64**	**PC65**
Standard deviation	0.003322	0.003278	0.003201	0.003144	0.003115
Proportion of Variance	0.00094	0.00092	0.00087	0.00084	0.00083
Cumulative Proportion	0.97031	0.97122	0.9721	0.97294	0.97376
	**PC66**	**PC67**	**PC68**	**PC69**	**PC70**
Standard deviation	0.003095	0.003027	0.002925	0.002871	0.002861
Proportion of Variance	0.00082	0.00078	0.00073	7.00E-04	7.00E-04
Cumulative Proportion	0.97458	0.97536	0.97609	0.97679	0.97749
	**PC71**	**PC72**	**PC73**	**PC74**	**PC75**
Standard deviation	0.002852	0.002757	0.002733	0.002713	0.002681
Proportion of Variance	0.00069	0.00065	0.00064	0.00063	0.00061
Cumulative Proportion	0.97818	0.97883	0.97947	0.98009	0.98071
	**PC76**	**PC77**	**PC78**	**PC79**	**PC80**
Standard deviation	0.002648	0.002546	0.0025	0.002483	0.002465
Proportion of Variance	6.00E-04	0.00055	0.00053	0.00053	0.00052
Cumulative Proportion	0.9813	0.98186	0.98239	0.98291	0.98343
	**PC81**	**PC82**	**PC83**	**PC84**	**PC85**
Standard deviation	0.002392	0.002356	0.00234	0.002301	0.002222
Proportion of Variance	0.00049	0.00047	0.00047	0.00045	0.00042
Cumulative Proportion	0.98392	0.98439	0.98486	0.98531	0.98573
	**PC86**	**PC87**	**PC88**	**PC89**	**PC90**
Standard deviation	0.002205	0.002199	0.002168	0.002152	0.002117
Proportion of Variance	0.00041	0.00041	4.00E-04	0.00039	0.00038
Cumulative Proportion	0.98615	0.98656	0.98696	0.98735	0.98773
	**PC91**	**PC92**	**PC93**	**PC94**	**PC95**
Standard deviation	0.002093	0.002053	0.002013	0.001968	0.001942
Proportion of Variance	0.00037	0.00036	0.00035	0.00033	0.00032
Cumulative Proportion	0.98811	0.98847	0.98881	0.98914	0.98946
	**PC96**	**PC97**	**PC98**	**PC99**	**PC100**
Standard deviation	0.001919	0.0019	0.001874	0.001826	0.001797
Proportion of Variance	0.00031	0.00031	3.00E-04	0.00028	0.00027
Cumulative Proportion	0.98978	0.99008	0.99038	0.99067	0.99094
	**PC101**	**PC102**	**PC103**	**PC104**	**PC105**
Standard deviation	0.001783	0.001758	0.001751	0.00172	0.001704
Proportion of Variance	0.00027	0.00026	0.00026	0.00025	0.00025
Cumulative Proportion	0.99121	0.99148	0.99174	0.99199	0.99224
	**PC106**	**PC107**	**PC108**	**PC109**	**PC110**
Standard deviation	0.001687	0.001661	0.001641	0.001634	0.001606
Proportion of Variance	0.00024	0.00024	0.00023	0.00023	0.00022
Cumulative Proportion	0.99248	0.99272	0.99295	0.99317	0.99339
	**PC111**	**PC112**	**PC113**	**PC114**	**PC115**

Standard deviation	0.001581	0.001555	0.001544	0.001541	0.001511
Proportion of Variance	0.00021	0.00021	2.00E-04	2.00E-04	0.00019
Cumulative Proportion	0.99361	0.99381	0.99401	0.99422	0.99441
	**PC116**	**PC117**	**PC118**	**PC119**	**PC120**
Standard deviation	0.001486	0.001469	0.001442	0.00144	0.001429
Proportion of Variance	0.00019	0.00018	0.00018	0.00018	0.00017
Cumulative Proportion	0.9946	0.99478	0.99496	0.99514	0.99531
	**PC121**	**PC122**	**PC123**	**PC124**	**PC125**
Standard deviation	0.001401	0.001388	0.001357	0.00134	0.001324
Proportion of Variance	0.00017	0.00016	0.00016	0.00015	0.00015
Cumulative Proportion	0.99548	0.99564	0.9958	0.99595	0.9961
	**PC126**	**PC127**	**PC128**	**PC129**	**PC130**
Standard deviation	0.001304	0.001284	0.001259	0.00125	0.001232
Proportion of Variance	0.00014	0.00014	0.00014	0.00013	0.00013
Cumulative Proportion	0.99625	0.99639	0.99652	0.99666	0.99678
	**PC131**	**PC132**	**PC133**	**PC134**	**PC135**
Standard deviation	0.001204	0.001196	0.001179	0.001168	0.001161
Proportion of Variance	0.00012	0.00012	0.00012	0.00012	0.00011
Cumulative Proportion	0.99691	0.99703	0.99715	0.99726	0.99738
	**PC136**	**PC137**	**PC138**	**PC139**	**PC140**
Standard deviation	0.001135	0.001121	0.001108	0.001095	0.001082
Proportion of Variance	0.00011	0.00011	1.00E-04	1.00E-04	1.00E-04
Cumulative Proportion	0.99749	0.9976	0.9977	0.9978	0.9979
	**PC141**	**PC142**	**PC143**	**PC144**	**PC145**
Standard deviation	0.001069	0.001052	0.001036	0.001031	0.001016
Proportion of Variance	1.00E-04	9.00E-05	9.00E-05	9.00E-05	9.00E-05
Cumulative Proportion	0.998	0.99809	0.99819	0.99828	0.99836
	**PC146**	**PC147**	**PC148**	**PC149**	**PC150**
Standard deviation	0.000998	0.000986	0.000971	0.000969	0.000958
Proportion of Variance	8.00E-05	8.00E-05	8.00E-05	8.00E-05	8.00E-05
Cumulative Proportion	0.99845	0.99853	0.99861	0.99869	0.99877
	**PC151**	**PC152**	**PC153**	**PC154**	**PC155**
Standard deviation	0.000946	0.000931	0.000903	0.000902	0.000879
Proportion of Variance	8.00E-05	7.00E-05	7.00E-05	7.00E-05	7.00E-05
Cumulative Proportion	0.99885	0.99892	0.99899	0.99906	0.99913
	**PC156**	**PC157**	**PC158**	**PC159**	**PC160**
Standard deviation	0.000868	0.000863	0.00085	0.000838	0.00083
Proportion of Variance	6.00E-05	6.00E-05	6.00E-05	6.00E-05	6.00E-05
Cumulative Proportion	0.99919	0.99925	0.99931	0.99937	0.99943
	**PC161**	**PC162**	**PC163**	**PC164**	**PC165**
Standard deviation	0.000822	0.000809	0.000803	0.00078	0.000769
Proportion of Variance	6.00E-05	6.00E-05	5.00E-05	5.00E-05	5.00E-05
Cumulative Proportion	0.99949	0.99955	0.9996	0.99965	0.9997
	**PC166**	**PC167**	**PC168**	**PC169**	**PC170**
Standard deviation	0.000751	0.000732	0.000722	0.000702	0.0007
Proportion of Variance	5.00E-05	5.00E-05	4.00E-05	4.00E-05	4.00E-05

Cumulative Proportion	0.99975	0.9998	0.99984	0.99988	0.99993
	**PC171**	**PC172**			
Standard deviation	0.000676	0.000648	3.27E-17		
Proportion of Variance	4.00E-05	4.00E-05	0		
Cumulative Proportion	0.99996	1	1		

Supplementary Table 2. Extended results of one-sided phylogenetic regressions, including interactions among factors (size, centroid size; eco, adult habitat; dev, larval feeding mode), test statistics and p-values for MCC tree, and percentage of trees from distribution of 100 trees randomly sampled in the posterior distribution with significant results. Significances based on permutations (n = 999). Text colour indicates significance of p < 0.01, 0.01 < p < 0.05, or p > 0.05.

	Pagel's λ		size	eco	dev	size:eco	size:dev	eco:dev	size:eco: dev
**Parasphenoid**	**0.54**	**Pillai's test**	0.92	4.24	0.77	4.38	0.67	4.23	3.52
		**p-value***	0.001	0.001	0.001	0.001	0.293	0.005	0.045
		**% trees sig**	100	100	100	99	0	96	45
		**SES**	7.51	7.02	3.49	4.46	0.55	2.76	1.58
**Squamosal**	**0.80**	**Pillai's test**	0.89	4.52	0.77	4.67	0.81	4.28	3.98
		**p-value***	0.001	0.001	0.001	0.001	0.001	0.001	0.001
		**% trees sig**	100	100	100	100	100	98	100
		**SES**	6.91	9.92	3.63	6.58	3.89	3.31	4.70
**Pterygoid**	**0.67**	**Pillai's test**	0.89	3.93	0.71	4.05	0.73	3.69	3.48
		**p-value***	0.001	0.001	0.001	0.001	0.004	0.149	0.002
		**% trees sig**	100	100	100	100	100	3	99
		**SES**	7.06	6.27	3.19	4.17	2.67	1.02	2.86
**Frontoparietal**	**0.20**	**Pillai's test**	0.94	4.49	0.83	4.48	0.82	4.44	3.86
		**p-value***	0.001	0.001	0.001	0.001	0.001	0.006	0.005
		**% trees sig**	100	100	100	99	99	98	98
		**SES**	7.85	8.17	4.06	4.28	3.32	2.85	2.82
**Nasal**	**0.63**	**Pillai's test**	0.81	3.70	0.49	3.77	0.47	3.26	2.68
		**p-value***	0.001	0.001	0.601	0.001	0.870	0.169	0.560
		**% trees sig**	100	100	1	100	0	5	1
		**SES**	7.21	7.77	0.30	4.64	1.15	0.91	0.17
**Otic**	**0.54**	**Pillai's test**	0.93	4.04	0.68	4.27	0.71	4.47	3.58
		**p-value***	0.001	0.001	0.043	0.001	0.044	0.001	0.004
		**% trees sig**	100	100	49	96	39	100	89
		**SES**	8.18	5.66	1.67	4.27	1.72	4.97	2.52
**Neopalatine**	**0.20**	**Pillai's test**	0.47	1.41	0.37	1.26	0.23	1.40	0.99
		**p-value***	0.001	0.163	0.009	0.901	0.619	0.554	0.968
		**% trees sig**	100	2	100	0	0	0	0
		**SES**	5.48	1.01	2.73	1.26	0.35	0.21	1.71
**Stapes**	**0.50**	**Pillai's test**	0.71	2.65	0.30	2.82	0.55	2.36	2.16
		**p-value***	0.001	0.001	0.092	0.001	0.001	0.001	0.001
		**% trees sig**	100	100	0	100	100	100	100
		**SES**	10.84	12.65	1.35	10.41	6.09	5.89	5.82
**Sphenethmoid(d)**	**0.72**	**Pillai's test**	0.60	2.12	0.33	2.07	0.33	1.92	1.85
		**p-value***	0.001	0.018	0.526	0.476	0.669	0.734	0.494
		**% trees sig**	100	94	2	7	0	0	0
		**SES**	5.66	2.10	0.08	0.05	0.49	0.69	0.04
**Vomer**	**0.70**	**Pillai's test**	0.62	2.03	0.45	1.80	0.35	1.90	1.90

		**p-value***	0.001	0.001	0.001	0.201	0.132	0.089	0.006
		**% trees sig**	100	100	100	3	4	15	94
		**SES**	7.78	4.53	3.84	0.87	1.14	1.37	2.92
**Sphenethmoid(v)**	**0.82**	**Pillai's test**	0.84	3.40	0.63	3.71	0.60	3.42	3.26
		**p-value***	0.001	0.001	0.002	0.001	0.012	0.001	0.001
		**% trees sig**	100	100	99	100	99	99	100
		**SES**	8.39	7.29	3.67	6.22	2.50	3.41	4.78
**Premaxilla**	**0.56**	**Pillai's test**	0.77	3.92	0.70	3.56	0.65	3.68	3.21
		**p-value***	0.001	0.001	0.002	0.089	0.033	0.018	0.016
		**% trees sig**	100	100	100	21	58	77	71
		**SES**	5.41	7.57	3.30	1.39	1.81	2.03	2.13
**Maxilla**	**0.62**	**Pillai's test**	0.92	4.85	0.81	4.92	0.82	4.68	4.13
		**p-value***	0.001	0.001	0.012	0.001	0.041	0.045	0.007
		**% trees sig**	100	100	98	99	52	26	99
		**SES**	5.67	7.22	2.13	4.25	1.68	1.68	2.65
**Occipital**	**0.50**	**Pillai's test**	0.97	4.86	0.85	4.89	0.81	4.88	4.16
		**p-value***	0.001	0.001	0.001	0.001	0.024	0.001	0.001
		**% trees sig**	100	100	100	100	91	3	99
		**SES**	7.50	8.34	3.72	4.96	1.93	4.47	3.57
**Quadratojugal**	**0.68**	**Pillai's test**	0.78	2.84	0.48	2.74	0.42	2.78	2.45
		**p-value***	0.001	0.001	0.012	0.001	0.268	0.004	0.014
		**% trees sig**	100	99	74	59	2	65	63
		**SES**	9.21	6.81	2.31	3.09	0.61	3.02	2.54
**Complete Skull**	**0.56**	**Pillai's test**	0.99	5.78	0.97	5.8	0.97	5.79	4.83
		**p-value***	0.001	0.001	0.001	0.001	0.003	0.001	0.002
		**SES**	3.58	5.78	2.5	3.54	1.85	2.93	2.12

Supplementary Table 3. Pairwise significance values for tests of differences in cranial disparity for the seven habitats, for each cranial region. Test statistics are the absolute difference in Procrustes variance between groups, with significance estimated with permutations (n = 999). Bold values indicate significance at the $p < 0.05$ level. Data are size-corrected. Disparity values for each region are shown in Supplementary Figure 8 and for the whole skull are detailed in Supplementary Note 1.

**Frontoparietal**	aquatic	arboreal	fossorial	semi-aquatic	semi-arboreal	semi-fossorial
aquatic						
arboreal	**0.01**					
fossorial	0.39	**0.04**				
semi-aquatic	**< 0.01**	0.15	**< 0.01**			
semi-arboreal	**0.02**	0.88	0.10	0.36		
semi-fossorial	0.09	0.74	0.27	0.18	0.68	
terrestrial	**< 0.01**	0.19	0.00	0.69	0.48	0.22
**Maxilla**	aquatic	arboreal	fossorial	semi-aquatic	semi-arboreal	semi-fossorial
aquatic						
arboreal	**0.01**					
fossorial	0.53	**0.03**				
semi-aquatic	**< 0.01**	0.36	**0.01**			
semi-arboreal	**0.01**	0.53	**0.03**	0.96		
semi-fossorial	0.74	**0.02**	0.35	**0.01**	**0.01**	
terrestrial	**< 0.01**	0.18	**< 0.01**	0.92	0.89	**0.01**
**Nasal**	aquatic	arboreal	fossorial	semi-aquatic	semi-arboreal	semi-fossorial
aquatic						
arboreal	**0.03**					
fossorial	0.57	0.10				
semi-aquatic	**< 0.01**	0.15	**0.01**			
semi-arboreal	**0.04**	0.74	0.12	0.46		
semi-fossorial	0.08	0.82	0.18	0.48	0.95	
terrestrial	**< 0.01**	**0.01**	**< 0.01**	0.68	0.28	0.30
**Neopalatine**	aquatic	arboreal	fossorial	semi-aquatic	semi-arboreal	semi-fossorial
aquatic						
arboreal	**< 0.01**					
fossorial	0.20	**0.02**				
semi-aquatic	**< 0.01**	0.92	**0.02**			
semi-arboreal	**0.01**	0.72	**0.04**	0.79		
semi-fossorial	0.27	0.05	0.99	0.07	0.08	
terrestrial	**< 0.01**	0.82	**0.01**	0.95	0.81	**0.04**
**Occipital**	aquatic	arboreal	fossorial	semi-aquatic	semi-arboreal	semi-fossorial
aquatic						

arboreal	**< 0.01**					
fossorial	0.24	**0.02**				
semi-aquatic	**< 0.01**	0.62	**0.02**			
semi-arboreal	**< 0.01**	0.57	**0.03**	0.83		
semi-fossorial	0.43	**0.04**	0.78	**0.04**	0.05	
terrestrial	**< 0.01**	0.33	**< 0.01**	0.77	0.98	**0.02**
**Otic**	aquatic	arboreal	fossorial	semi-aquatic	semi-arboreal	semi-fossorial
aquatic						
arboreal	0.33					
fossorial	0.30	**0.03**				
semi-aquatic	0.12	0.39	**0.01**			
semi-arboreal	0.23	0.56	**0.05**	0.92		
semi-fossorial	0.11	**0.03**	0.39	**0.01**	**0.03**	
terrestrial	0.27	0.91	**0.02**	0.38	0.60	**0.02**
**Parasphenoid**	aquatic	arboreal	fossorial	semi-aquatic	semi-arboreal	semi-fossorial
aquatic						
arboreal	**< 0.01**					
fossorial	**< 0.01**	0.15				
semi-aquatic	**< 0.01**	0.39	0.07			
semi-arboreal	**< 0.01**	0.71	0.18	0.76		
semi-fossorial	**< 0.01**	0.69	0.46	0.35	0.47	
terrestrial	**< 0.01**	0.63	0.05	0.68	0.98	0.47
**Premaxilla**	aquatic	arboreal	fossorial	semi-aquatic	semi-arboreal	semi-fossorial
aquatic						
arboreal	0.36					
fossorial	0.73	0.14				
semi-aquatic	0.05	0.13	**0.01**			
semi-arboreal	0.20	0.53	0.09	0.66		
semi-fossorial	0.32	0.05	0.43	**0.01**	**0.04**	
terrestrial	0.09	0.24	**0.01**	0.52	0.99	**0.01**
**Pterygoid**	aquatic	arboreal	fossorial	semi-aquatic	semi-arboreal	semi-fossorial
aquatic						
arboreal	**< 0.01**					
fossorial	0.47	**< 0.01**				
semi-aquatic	**< 0.01**	0.57	**< 0.01**			
semi-arboreal	**0.01**	0.55	**0.01**	0.94		
semi-fossorial	0.11	0.14	0.19	0.11	0.14	
terrestrial	**< 0.01**	0.48	**< 0.01**	1.00	0.92	0.06

**Quadratojugal**	aquatic	arboreal	fossorial	semi-aquatic	semi-arboreal	semi-fossorial
aquatic						
arboreal	0.30					
fossorial	**0.01**	**< 0.01**				
semi-aquatic	0.09	0.33	**< 0.01**			
semi-arboreal	0.14	0.42	**< 0.01**	0.99		
semi-fossorial	**0.02**	**< 0.01**	0.68	**< 0.01**	**< 0.01**	
terrestrial	0.17	0.68	**< 0.01**	0.47	0.60	**< 0.01**

**Sphenethmoid dorsal**	aquatic	arboreal	fossorial	semi-aquatic	semi-arboreal	semi-fossorial
aquatic						
arboreal	**0.02**					
fossorial	0.12	0.50				
semi-aquatic	**0.01**	0.31	0.13			
semi-arboreal	**0.03**	0.58	0.30	0.85		
semi-fossorial	0.29	0.44	0.87	0.14	0.26	
terrestrial	**0.01**	0.89	0.34	0.34	0.62	0.37

**Sphenethmoid ventral**	aquatic	arboreal	fossorial	semi-aquatic	semi-arboreal	semi-fossorial
aquatic						
arboreal	**< 0.01**					
fossorial	**0.02**	0.16				
semi-aquatic	**< 0.01**	0.26	**0.03**			
semi-arboreal	**< 0.01**	0.19	**0.04**	0.70		
semi-fossorial	**0.01**	0.92	0.30	0.53	0.38	
terrestrial	**< 0.01**	0.82	0.09	0.31	0.23	0.99

**Squamosal**	aquatic	arboreal	fossorial	semi-aquatic	semi-arboreal	semi-fossorial
aquatic						
arboreal	0.15					
fossorial	**0.03**	**< 0.01**				
semi-aquatic	0.08	0.52	**< 0.01**			
semi-arboreal	0.13	0.69	**< 0.01**	0.88		
semi-fossorial	0.19	**0.04**	0.34	**0.02**	**0.04**	
terrestrial	0.09	0.82	**< 0.01**	0.61	0.79	**0.03**

**Stapes**	aquatic	arboreal	fossorial	semi-aquatic	semi-arboreal	semi-fossorial
aquatic						
arboreal	**0.04**					
fossorial	0.80	**0.02**				
semi-aquatic	0.12	0.75	0.06			
semi-arboreal	0.07	0.67	0.04	0.55		

semi-fossorial	0.84	0.15	0.70	0.26	0.15	
terrestrial	0.21	0.24	0.11	0.49	0.27	0.44
**Vomer**	aquatic	arboreal	fossorial	semi-aquatic	semi-arboreal	semi-fossorial
aquatic						
arboreal	0.32					
fossorial	**0.04**	**< 0.01**				
semi-aquatic	0.20	0.55	**< 0.01**			
semi-arboreal	0.46	0.86	**0.01**	0.51		
semi-fossorial	0.97	0.31	0.08	0.21	0.42	
terrestrial	0.28	0.99	**< 0.01**	0.55	0.88	0.28
**Whole cranium**	aquatic	arboreal	fossorial	semi-aquatic	semi-arboreal	semi-fossorial
aquatic						
arboreal	**< 0.01**					
fossorial	**< 0.01**	**< 0.01**				
semi-aquatic	**0.01**	**< 0.01**	**0.01**			
semi-arboreal	**0.01**	**< 0.01**	**0.01**	**< 0.01**		
semi-fossorial	**< 0.01**	**< 0.01**	**< 0.01**	**0.01**	**0.01**	
terrestrial	**0.01**	**< 0.01**	**0.01**	**< 0.01**	**< 0.01**	**0.01**

Supplementary Table 4. Results for differences in cranial disparity according to larval feeding mode for each cranial region. Disparity is corrected for semi/landmark number. Test statistics are the absolute difference in Procrustes variance between groups, with significance estimated with permutations (n = 999). Bold values indicate significance at the $p < 0.05$ level. Data are size-corrected.

**Cranial region**	**Disparity of taxa with feeding larvae/ 10^{-4}**	**Disparity of taxa with non-feeding larvae / 10^{-4}**	**Significance (*p* value)**
Frontoparietal	9.68	10.75	0.60
Maxilla	16.18	19.45	0.29
Nasal	5.45	4.85	0.50
Neopalatine	2.57	4.15	0.07
Occipital	6.43	8.07	0.37
Otic	6.49	13.53	**< 0.01**
Parasphenoid	4.66	4.29	0.78
Premaxilla	4.82	7.23	**0.02**
Pterygoid	7.67	6.74	0.56
Quadratojugal	7.30	7.07	0.88
Sphenethmoid (dorsal)	3.23	4.54	0.05
Sphenethmoid (ventral)	3.74	5.38	**0.04**
Squamosal	12.83	16.71	0.26
Stapes	2.58	3.79	0.12
Vomer	1.43	3.12	**0.01**
Whole cranium	0.10	0.12	0.105

Supplementary Table 5. Definition of the 15 cranial regions in this study.

Cranial regions	Definition	Position when absent
**Occipital and Otic regions**	The opisthotic fuses with the exoccipital through development[2], forming the oto-occipital bone. The prootic is variably fused to the oto-occipital bone[2,3]. When the prootic is fused indistinguishably with the oto-occipital bone, it is not possible to determine the precise location of the original sutures. However, these bones could not be placed into one region, since they are separated by unossified tissue in many specimens[3]. Furthermore, these bones likely have distinct functions (the oto-occipital is involved in cranial-neck articulation and the prootic is more invested in the otic region). The separation between the oto-occipital and prootic occurs in the dorsal region of the otic capsule[3], along or near the posterior epiotic ridge. Therefore, when necessary, we split the otoccipital region along this ridge, forming an 'occipital' region corresponding primarily to the exoccipital (and opisthotic), and an 'otic' region corresponding primarily to the prootic. This created distinct 'back-of-skull' and 'otic' functional regions.<br><br>**Occipital**<br><br>'Back-of-skull' region, mostly corresponding to exoccipital and opisthotic bones. Curves were traced around the jugular foramen. This region includes the occipital condyle.<br><br>**Otic**<br><br>'Otic' region, most comprising the prootic. This region extends ventrally until the prootic foramen. Medially, this region is defined by a curve tracing the medial border of the epiotic eminence. In *Brachycephalus ephippium*, this region includes the parotic plate.	NA
**Frontoparietal**	External surface of frontoparietal, which for most specimens is simply the dorsal surface. However, the lateral margin of the frontoparietal can reach far ventrally (e.g., *Peltophryne guentheri*). Capturing the lateral surface of the frontoparietal allowed depth information to be captured, including the tall ridge present in some specimens (particularly hyperossified frogs, which typically have extremely dorsoventrally tall frontoparietals). A frontoparietal-squamosal arch ('arcus postorbitalis') can also be present in hyperossified frogs, where the lateral process of the frontoparietal extends far laterally and sutures with the squamosal, overlying the otic region. In these cases, the curves defining the frontoparietal pass underneath this arch, along the suture of the frontoparietal with the otic region. In extremely ossified taxa with the posterolateral margin of the frontoparietal not visible, we placed this curve immediately medial to the anterior epiotic ridge, separating the frontoparietal from the otic region. The frontoparietal is a paired bone in most specimens. When unpaired, half of the bone is captured.	NA
**Maxilla (lateral and ventral)**	Lateral surface of maxilla, extending ventrally until reaching (but excluding) tooth row and palatal shelf (palatine process) of maxilla, posteromedial to the 'pars dentalis'.	NA
**Nasal**	Exposed surface of nasal. The posterolateral border of this region traces the posterolateral margin of the nasal, so that the dorsoventral thickness of this bone is captured.	NA
**Neopalatine and Vomer**	The vomer and neopalatine can fuse in some specimens to form the vomeropalatine (e.g., *Xenorhina* sp.). In this case, the vomeropalatine was split into two regions which best divided this bone into the neopalatine and vomer regions. Specifically, the posterior process	

	occupying the position of the neopalatine in other frogs was defined as the neopalatine region. The neopalatine and vomer are also variably fused with the sphenethmoid. When the original sutures were not discernable, osteological information from different species within that genus was used. For example, *Atelopus oxyrynchus* was landmarked as having a vomer and palatine because other *Atelopus* species do, and the region boundaries were inferred from other species.<br><br>**Neopalatine**<br><br>Ventral surface of neopalatine. In the rare case that teeth are present, these are manually removed. This region is absent in some specimens.<br><br>**Vomer**<br><br>Ventral surface of vomer, including anterior, prechoanal, postchoanal and dentigerous processes. The vomerine teeth in most specimens are situated on a ridge on the posterior margin of the vomer. In these cases, our defined region included the tooth ridge, but excluded the teeth themselves (e.g., *Agalychnis*). When teeth were situated in the middle and not the edge of the vomer, teeth were manually removed so that the entire surface of the vomer could still be captured (*Kassina arboricola, Spea multiplicata, Rhacophorus reinwalti, Chacophrys pierotti*). The only exception to this was when occasionally a small area of bone was present posterior to the tooth ridge of the vomer, but the teeth were difficult to manually remove (*Oreobates quixensis, Discodeles bufoniformis, Polypedates macrotis*). In these cases this posteriorly located bone was excluded from the region.	**Neopalatine:**<br><br>Posteromedial extreme of vomer. If vomer is also absent: anteromedial tip of the parasphenoid (e.g., *Brachycephalus ephippium*).<br><br>**Vomer:**<br><br>Anteromedial tip of parasphenoid (e.g., *Pipa pipa*)
**Parasphenoid**	The exposed surface of the parasphenoid. The pterygoid can variably overlie the parasphenoid, so that the parasphenoid curves trace around the pterygoid. For specimens where the parasphenoid has fused indistinguishably to the sphenethmoid (e.g., *Xenopus laevis*), the lateral margin of the parasphenoid region was taken as the extreme of the ventral surface of fused bone.	NA
**Premaxilla (dorsal and ventral)**	Anterior surface of premaxilla (dorsal to 'pars dentalis'), including alary process, and the palatine shelf ('pars palatina') of premaxilla, including the two posterior processes present in most specimens, but excluding the 'pars dentalis' (tooth shelf).	NA
**Pterygoid**	Ventral surface of pterygoid, capturing anterior ramus, medial ramus and posterior ramus morphology.	NA
**Quadratojugal (jaw joint and lateral)**	The quadratojugal region comprises two surfaces of the quadratojugal: the jaw joint articular surface and the lateral process.<br><br>**Jaw joint:**<br><br>The quadrate is cartilaginous, and ossifies only in some species (e.g., the ascaphids, pelobatids, and *Brachycephalus*)[4]. It is often invaded by the ossified quadratojugal[4]. When no ossified material is present we class this surface as absent. When it is partially ossified, we capture as much of this surface as possible. The quadratojugal articulates with the pterygoid and the mandible[5]. The jaw joint articular surface was often adjacent to the pterygoid articular surface, so to identify the jaw joint articular surface we referred to reconstructions of each skull in situ with the mandible.<br><br>**Lateral process:**<br><br>We defined the lateral surface of the quadratojugal as the maxillary process. Only the exposed surface of the maxillary process of the	**Jaw joint:**<br><br>Posteroventral extreme of the lateral surface of the quadrate (e.g., *Limnomedusa macroglossa*). When the lateral surface of the quadrate is also absent, the negligible region is placed on the anterolateral tip of the squamosal.<br><br>**Lateral process:**

	quadratojugal is captured, so that this area is marked as absent in *Triprion* as in this species it is covered by the overlying maxilla.<br><br>If either the jaw joint or lateral surface was absent in a specimen, then that surface was classed as absent and the remaining surface was captured as usual.	Anterolateral tip of the squamosal.
**Sphenethmoid (dorsal)**	This region captures the ossified region medial to the paired nasals or frontoparietals. This region is the dorsal surface of the sphenethmoid in most anurans, or the dermal sphenethmoid in some frogs (e.g., casque-headed hylids[4]), as the dermal sphenethmoid overlies the dorsal sphenethmoid. This ossified region can either be paired (e.g., *Alytes obstetricans*) or not (e.g., *Bombina maxima*), where half of the region is captured in the latter case.	Anteromedial extreme of frontoparietal (e.g., *Eleutherodactylus bicorpatus*).
**Sphenethmoid (ventral)**	The exposed, ossified surface of the sphenethmoid in ventral and lateral aspects. At its largest, this region can extend anteriorly between the paired vomers, and it can extend far posteriorly, lateral to the parasphenoid. The extent of ossification varies across specimens as follows: ossified both anterior and lateral to the parasphenoid (most specimens, e.g. *Hylodes perplicatus*), only anterior to the parasphenoid (e.g. *Rhinoderma darwinii*), only lateral to the parasphenoid (e.g. *Atelognathus patagonicus*), or completely unossified, and hence lacking this region (e.g. *Caudiverbera caudiverbera*). When there was no natural margin for which to limit this region posteriorly, we defined the posterior limit as the anterior margin of the optic foramen. The orbitonasal foramen was manually filled in for all specimens, as this was either entirely or partially enclosed by the ventral sphenethmoid. When the ventral surface of the sphenethmoid is partially covered by the overlapping vomer, we captured only the surface that was accessible.	Anteromedial tip of parasphenoid.
**Squamosal**	The twisting nature of the squamosal bone in the crania of most frogs hinders the simple delimitation of this region. The squamosal morphology is captured in dorsal and lateral aspect, to best define the external surface of this bone. Specifically, the zygomatic (anterior) ramus and otic (posterior) ramus are captured in dorsal aspect, and the ventral ramus in lateral aspect. In rare cases when a ramus is absent, the landmarks and curves are placed bunched together in the position that best represents where the ramus would have been.	NA
**Stapes**	Lateral surface.	Posterior extreme position on margin of fenestra ovalis

Supplementary Table 6. Landmark definitions used in this study. 82 landmarks were placed onto the right-hand side of the cranium of each specimen. 24 landmarks (*) were removed prior to analyses as they were not homologous across all specimens. These 24 landmarks were used only in the patching procedure to place surface points onto variably present regions.

Original landmark number	Landmark definition	Cranial region
1	Premaxilla: medial extreme above tooth row	Premaxilla
2	Premaxilla: lateral extreme above tooth row	Premaxilla
3	Premaxilla: tip of alary process ('pars alaris')	Premaxilla
4	Premaxilla: posteromedial extreme at base of alary process	Premaxilla
5	Premaxilla: posterolateral extreme at base of alary process	Premaxilla
6	Maxilla: anteroventral extreme in lateral view, dorsal to the tooth row/palatal shelf	Maxilla
7	Maxilla: posterior extreme in lateral aspect.	Maxilla
8	Maxilla: anterodorsal extreme of maxilla in lateral view.	Maxilla
9	Maxilla: tip of preorbital process of pars facialis, or if process absent, posterior-most suture with nasal	Maxilla
10	Parasphenoid: anterior tip of cultriform process	Parasphenoid
11	Parisphenoid: inflection point between anterior and lateral projections	Parasphenoid
12	Parisphenoid: anterolateral extreme of lateral projection	Parasphenoid
13	Parisphenoid: inflection point between lateral and posterior projections	Parasphenoid
14	Parasphenoid: posterior extreme along midline	Parasphenoid
15	Squamosal: posteromedial extreme on tip of otic (posterior) ramus	Squamosal
16	Squamosal: medial extreme on tip of ventral ramus	Squamosal
17	Squamosal: ventral extreme on tip of zygomatic (anterior) ramus	Squamosal
18	Squamosal: inflection point between zygomatic (anterior) ramus and ventral ramus	Squamosal
19	Squamosal: anterolateral tip of ventral ramus	Squamosal
20	Squamosal: inflection point between ventral ramus and otic (posterior) ramus	Squamosal
21	Pterygoid: lateral extreme of tip of posterior ramus	Pterygoid
22	Pterygoid: inflection point between medial ramus and posterior ramus on the ventral bone surface	Pterygoid
23	Pterygoid: anteromedial extreme of tip of medial ramus	Pterygoid
24	Pterygoid: anterior extreme of base of medial ramus	Pterygoid
25	Pterygoid: anteromedial extreme of anterior ramus	Pterygoid
26	Pterygoid: anterior extreme of base of posterior ramus	Pterygoid
27	Frontoparietal: anterior extreme along medial margin	Frontoparietal
28	Frontoparietal: anterior extreme along lateral margin	Frontoparietal
29	Frontoparietal: most lateral suture with otic region	Frontoparietal
30	Frontoparietal: posterolateral extreme, often adjacent to epiotic ridge	Frontoparietal
31	Frontoparietal: suture with exoccipital along midline	Frontoparietal
32	Premaxilla: anteromedial extreme position on palatal shelf ('pars palatina') (just posterior to the pars dentigera)	Premaxilla
33	Premaxilla: anterolateral extreme position on palatal shelf ('pars palatina') (just posterior to the pars dentigera)	Premaxilla
34	Premaxilla: posterolateral extreme of palatal shelf ('pars palatina')	Premaxilla
35	Premaxilla: tip of palatine process on palatal shelf ('pars palatina'), in ventral aspect	Premaxilla
36	Maxilla: anterior extreme of palatal shelf ('pars palatina')	Maxilla
37	Maxilla: anteromedial extreme of palatal shelf ('pars palatina')	Maxilla

38	Maxilla: posterolateral extreme of palatal shelf ('pars palatina')	Maxilla
39	Nasal: anterior tip	Nasal
40	Nasal: posterior extreme	Nasal
41	Nasal: tip of maxillary process	Nasal
42	*(Deleted landmark)**	*NA*
43	Parasphenoid: posterolateral extreme of lateral projection	Parasphenoid
44	Occipital: anteromedial extreme suture with frontoparietal	Occipital
45	Occipital: dorsal extreme of foramen magnum	Occipital
46	Occipital: dorsal extreme of occipital condyle, along foramen magnum margin	Occipital
47	Occipital: ventral extreme of occipital condyle, along foramen magnum margin	Occipital
48	Occipital: ventral extreme of foramen magnum	Occipital
49	Occipital: in ventral aspect, posteromedial suture with parasphenoid	Occipital
50	Stapes: dorsolateral extreme*	Stapes
51	Stapes: ventrolateral extreme*	Stapes
52	Stapes: dorsomedial extreme*	Stapes
53	Stapes: ventromedial extreme*	Stapes
54	Vomer: tip of anterior process (or anterior extreme)*	Vomer
55	Vomer: tip of prechoanal process (or anterolateral extreme)*	Vomer
56	Vomer: tip of postchoanal process (or posterolateral extreme)*	Vomer
57	Vomer: posteromedial extreme*	Vomer
58	Occipital: in ventral aspect, posterolateral extreme suture with parasphenoid	Occipital
59	Occipital: ventral extreme of jugular foramen	Occipital
60	Occipital: dorsal extreme of jugular foramen	Occipital
61	Occipital: anterior extreme of epiotic ridge	Occipital
62	Occipital: juncture of epiotic ridge and posterior margin of otic region	Occipital
63	Otic region: anterior extreme of epiotic ridge	Otic
64	Otic region: dorsolateral extreme of prootic foramen	Otic
65	Otic region: anterolateral extreme, following anterior margin of otic region	Otic
66	Otic region: posterolateral extreme	Otic
67	Otic region: juncture of epiotic ridge and posterior margin of otic region	Otic
68	Quadratojugal: articular surface, posterolateral extreme on hinge*	Quadratojugal
69	Quadratojugal: articular surface, anterior extreme*	Quadratojugal
70	Quadratojugal: articular surface, posteromedial extreme on hinge*	Quadratojugal
71	Quadratojugal: maxillary process, anterodorsal tip*	Quadratojugal
72	Quadratojugal: maxillary process, posterodorsal extreme*	Quadratojugal
73	Quadratojugal: maxillary process, posteroventral extreme*	Quadratojugal
74	Neopalatine: medial tip*	Neopalatine
75	Neopalatine: anterior extreme of lateral tip*	Neopalatine
76	Neopalatine: posterior extreme of lateral tip*	Neopalatine
77	Sphenethmoid (dorsal): anteromedial extreme*	Sphenethmoid (dorsal)
78	Sphenethmoid (dorsal): posteromedial extreme*	Sphenethmoid (dorsal)
79	Sphenethmoid (dorsal): lateral extreme*	Sphenethmoid (dorsal)
80	Sphenethmoid (ventral): anteromedial extreme*	Sphenethmoid (ventral)

81	Sphenethmoid (ventral): posteromedial extreme, just anterior to the optic foramen*	Sphenethmoid (ventral)
82	Sphenethmoid (ventral): posterolateral extreme*	Sphenethmoid (ventral)
83	Sphenethmoid (ventral): anterolateral extreme, posterior to neopalatine when present*	Sphenethmoid (ventral)

Supplementary Table 7. Curve definitions used in this study. Curves are placed manually on each specimen and then converted into eight or nine curve points after they are exported from Landmark Editor, and then we subsampled curve points to a density reflecting the magnitude of variation along each curve. Curve points are then slid to minimise bending energy across the dataset. Some curves were placed for use in patching the variably-present regions, but were removed prior to analyses as these curves were not present across the entire dataset. C#: curve number; C_n#: new curve number; LM_{st}: starting landmark; LM_{en}: ending landmark; CL_{MP}: manually-placed curve length; CL_R: resampled curve length.

C#	C_n#	Description	Cranial region	LM_{st}	LM_{en}	CL_{MP}	CL_R
C1	C1	Directly to the base of the alary process ('pars alaris') then follow base	Premaxilla	1	4	3	3
C2	C2	Following posterior edge of alary process ('pars alaris')	Premaxilla	4	3	4	3
C3	C3	Following posterior edge of alary process ('pars alaris')	Premaxilla	3	5	4	3
C4	C4	Following base of alary process ('pars alaris'), then directly to landmark	Premaxilla	5	2	3	3
C5	C5	Medially, dorsal to tooth row	Premaxilla	2	1	4	4
C6	C6	Laterally, posterior to tooth shelf ('pars dentigera')	Premaxilla	32	33	4	3
C7	C7	Following edge of palatal shelf of premaxilla	Premaxilla	33	34	3	3
C8	C8	Following edge of palatal shelf of premaxilla	Premaxilla	34	35	4	6
C9	C9	Following edge of palatal shelf of premaxilla	Premaxilla	35	32	4	3
C10	C10	Following bone margin	Parasphenoid	10	11	10	10
C11	C11	Following bone margin	Parasphenoid	11	12	5	6
C12	C12	Following bone margin	Parasphenoid	12	43	6	4
C13	C13	Following bone margin	Parasphenoid	43	13	5	5
C14	C14	Following bone margin	Parasphenoid	13	14	3	4
C15	C15	Anteriorly along midline	Parasphenoid	14	10	8	12
C16	C16	Following margin of ventral bone surface	Pterygoid	21	22	6	5
C17	C17	Following margin of ventral bone surface	Pterygoid	22	23	6	5
C18	C18	Following margin of ventral bone surface	Pterygoid	23	24	5	5
C19	C19	Following margin of ventral bone surface	Pterygoid	24	25	8	8
C20	C20	Following margin of ventral bone surface	Pterygoid	25	26	10	10
C21	C21	Following margin of ventral bone surface	Pterygoid	26	21	6	5
C22	C22	Posteriorly along margin of palatal shelf, excluding tooth shelf	Maxilla	36	38	10	15
C23	C23	Anteriorly along margin of palatal shelf	Maxilla	38	37	12	15
C24	C24	Laterally along margin	Maxilla	37	36	3	5
C25	C25	Tracing medial margin of dorsal surface	Squamosal	15	17	8	10

C26	C26	Following margin of lateral bone surface	Squamosal	17	18	6	10
C27	C27	Following margin of lateral bone surface	Squamosal	18	19	6	10
C28	C28	Following bone margin	Squamosal	19	16	3	5
C29	C29	Following medial extreme of the lateral bone surface in posterior view	Squamosal	16	20	6	8
C30	C30	Tracing bone margin to capture dorsal bone surface	Squamosal	20	15	6	10
C31	C31	Anteriorly along margin	Frontoparietal	27	28	4	5
C32	C32	Posteriorly along margin	Frontoparietal	28	29	8	15
C33	C33	Posteriorly along margin	Frontoparietal	29	30	5	5
C34	C34	Medially along margin	Frontoparietal	30	31	6	5
C35	C35	Anteriorly along margin	Frontoparietal	31	27	10	10
C36	*NA*	Following anterior margin	Neopalatine	74	75	6	5
C37	*NA*	Following lateral margin	Neopalatine	75	76	3	2
C38	*NA*	Following posterior margin	Neopalatine	76	74	6	5
C39	*NA*	Following lateral margin, in lateral view	Stapes	50	51	2	2
C40	*NA*	Following ventral margin, in lateral view	Stapes	51	53	4	5
C41	*NA*	Following medial margin, in lateral view	Stapes	53	52	3	3
C42	*NA*	Following dorsal margin, in lateral view	Stapes	52	50	4	5
C43	C36	Following midline to foramen magnum	Occipital	44	45	3	3
C44	C37	Tracing foramen magnum	Occipital	45	46	5	5
C45	C38	Tracing lateral margin of occipital condyle	Occipital	46	47	6	5
C46	C39	Tracing medial margin of occipital condyle	Occipital	46	47	4	4
C47	C40	Tracing foramen magnum	Occipital	47	48	3	3
C48	C41	Anteriorly along bone margin, or along midline if fused medially	Occipital	48	49	3	3
C49	C42	Laterally, tracing bine margin	Occipital	49	58	6	5
C50	C43	Trace around posterior margin of fenestra ovalis, to the ventral extreme of the epiotic ridge, and then dorsally along ridge.	Occipital	58	62	8	10
C51	C44	Tracing along ridge dorsally	Occipital	62	61	6	5
C52	C45	Medially tracing along bone margin	Occipital	61	44	5	5
C53	C46	Laterally around jugular foramen	Occipital	60	59	5	5
C54	C47	Medially around jugular foramen	Occipital	59	60	5	5
C55	C48	Anteriorly, tracing medial side of ridge	Otic	63	64	6	8
C56	C49	Laterally, following contour	Otic	64	65	5	5
C57	C50	Posteriorly, tracing lateral margin of dorsal bone surface	Otic	65	66	5	15

C58	C51	Medially, following contour	Otic	66	67	5	8
C59	C52	Tracing along epiotic ridge	Otic	67	63	5	5
C60	C53	Posteriorly tracing bone margin	Nasal	39	40	7	10
C61	C54	Laterally tracing bone margin, capturing bone depth	Nasal	40	41	7	10
C62	C55	Anteriorly tracing bone margin	Nasal	41	39	8	10
C63	*NA*	Posteriorly tracing bone margin	Vomer	54	55	8	6
C64	*NA*	Posteriorly tracing bone margin	Vomer	55	56	8	6
C65	*NA*	Tracing posterior bone margin, onto tooth ridge (if present) but anterior to teeth	Vomer	56	57	8	8
C66	*NA*	Anteriorly tracing bone margin	Vomer	57	54	8	6
C67	*NA*	Posteriorly, tracing bone margin	Quadratojugal	71	72	6	5
C68	*NA*	Ventrally, tracing bone margin	Quadratojugal	72	73	4	3
C69	*NA*	Anteriorly, tracing bone margin	Quadratojugal	73	71	6	5
C70	*NA*	Anteriorly, tracing articular surface only	Quadratojugal	68	69	4	5
C71	*NA*	Posteriorly, tracing articular surface only	Quadratojugal	69	70	4	5
C72	*NA*	Laterally, tracing articular surface only	Quadratojugal	70	68	4	5
C73	C56	Posteriorly, tracing bone margin	Maxilla	9	7	12	15
C74	C57	Anteriorly, tracing bone margin dorsal to tooth row	Maxilla	7	6	12	15
C75	C58	Dorsally, tracing bone margin	Maxilla	6	8	4	5
C76	C59	Posteriorly, tracing bone margin	Maxilla	8	9	6	8
C77	*NA*	Posteriorly, following midline	Sphenethmoid (dorsal)	77	78	5	8
C78	*NA*	Laterally, tracing bone margin	Sphenethmoid (dorsal)	78	79	5	8
C79	*NA*	Anteriorly, tracing bone margin	Sphenethmoid (dorsal)	79	77	8	10
C80	*NA*	Posteriorly, following midline	Sphenethmoid (ventral)	80	81	8	10
C81	*NA*	Dorsally, tracing posterior margin (or tracing anteriorly around optic foramen if bone extends posteriorly to this foramen)	Sphenethmoid (ventral)	81	82	4	5
C82	*NA*	Tracing ventral bone surface	Sphenethmoid (ventral)	82	83	8	10
C83	*NA*	Tracing ventral bone surface	Sphenethmoid (ventral)	83	80	10	15

Supplementary Table 8. Number of surface points within each of the 15 cranial regions. Surface points were distributed evenly over each region. Two templates were required for some regions (*), as one template was not sufficient to successfully patch the regions across all specimens owing to the extreme morphological variation across the dataset.

**Cranial region**	**Number of surface points**
Premaxilla	24
Parasphenoid*	40
Pterygoid	26
Maxilla	68
Nasal	20
Occipital	70
Otic	36
Frontoparietal*	64
Squamosal	26
Neopalatine	16
Stapes	16
Quadratojugal	31
Sphenethmoid (dorsal)*	29
Vomer	20
Sphenethmoid (ventral)*	41
**TOTAL**	**527**

Supplementary Table 9. Centroid size ('Csize') for each specimen

Species	Csize	Species	Csize	Species	Csize	Species	Csize
Acanthixalus sonjae	237.72	*Ceuthomantis smaragdinus*	134.38	*Indirana gundia*	239.06	*Raorchestes* sp.	162.32
Adenomera andreae	164.23	*Chacophrys pierottii*	296.86	*Kassina arboricola*	221.03	*Phlyctimantis boulengeri*	259.43
Adenomus kelaartii	224.86	*Chiromantis rufescens*	270.11	*Lankanectes corrugatus*	251.26	*Phrynobatrachus leveleve*	107.95
Afrixalus aureus	115.68	*Chrysobatrachus cupreonitens*	139.73	*Leiopelma hamiltoni*	314.57	*Phyllomedusa azurea*	199.03
Cruziohyla calcarifer	399.60	*Cochranella granulosa*	157.39	*Leptobrachium hasseltii*	385.84	*Pipa parva*	182.19
Allobates kingsburyi	125.65	*Conraua beccarii*	719.35	*Leptodactylodon boulengeri*	251.60	*Pipa pipa*	684.80
Allophryne ruthveni	127.66	*Conraua crassipes*	358.00	*Leptodactylus melanonotus*	242.71	*Platymantis polillensis*	162.72
Alsodes nodosus	304.76	*Conraua goliath*	1550.81	*Leptodactylus podicipinus*	217.43	*Plethodontohyla notosticta*	184.04
Altiphrynoides malcolmi	189.81	*Albericus darlingtoni*	147.09	*Leptopelis ocellatus*	308.55	*Polypedates macrotis*	387.11
Alytes obstetricans	270.51	*Craugastor laticeps*	363.91	*Leptopelis spiritusnoctis*	283.40	*Poyntonia paludicola*	160.25
Ameerega parvula	146.92	*Crossodactylus trachystomus*	144.71	*Limnomedusa macroglossa*	328.62	*Pristimantis chiastonotus*	304.38
Sclerophrys regularis	371.85	*Cryptobatrachus boulengeri*	244.82	*Limnonectes macrocephalus*	593.85	*Probreviceps macrodactylus*	274.68
Anaxyrus fowleri	284.90	*Cryptothylax greshoffii*	262.53	*Rana clamitans*	384.28	*Proceratophrys boiei*	376.38
Anaxyrus quercicus	185.94	*Cycloramphus asper*	382.78	*Litoria* sp.	342.27	*Pseudacris ornata*	188.48
Anhydrophryne rattrayi	114.67	*Cyclorana longipes*	440.07	*Mannophryne herminae*	205.54	*Pseudis paradoxa*	397.48
Anotheca spinosa	390.13	*Dendrobates* sp.	122.40	*Mantella baroni*	136.16	*Ptychadena bibroni*	317.17
Ansonia mcgregori	241.60	*Discodeles bufoniformis*	1019.39	*Melanobatrachus indicus*	171.44	*Pyxicephalus adspersus*	722.04
Aromobates alboguttatus	176.54	*Duttaphrynus dodsoni*	277.19	*Melanophryniscus stelzneri*	142.26	*Fejervarya vittigera*	251.55
Arthroleptella lightfooti	97.80	*Strabomantis cornutus*	304.52	*Mertensophryne micranotis*	111.76	*Fejervarya cancrivora*	451.26
Arthroleptis bioko	189.84	*Strabomantis biporcatus*	240.03	*Microbatrachella capensis*	100.86	*Rana sylvatica*	236.96
Ascaphus truei	233.25	*Eleutherodactylus glaphycompus*	223.67	*Minervarya nilagirica*	240.19	*Raorchestes* sp.	219.68
Astylosternus diadematus	333.78	*Eleutherodactylus johnstonei*	166.31	*Morerella cyanophthalma*	195.82	*Rhacophorus reinwardtii*	365.67
Astylosternus occidentalis	402.96	*Eupsophus roseus*	231.30	*Myobatrachus gouldii*	179.33	*Rheobatrachus silus*	267.76
Atelopus ignescens	198.07	*Fritziana goeldii*	247.60	*Nasikabatrachus sahyadrensis*	339.52	*Rhinoderma darwinii*	155.93
Atelopus oxyrhynchus	228.84	*Gastrophryne carolinensis*	117.41	*Natalobatrachus bonebergi*	205.93	*Rhinophrynus dorsalis*	230.76
Balebreviceps hillmani	177.80	*Gastrotheca peruana*	295.67	*Nectophrynoides tornieri*	169.97	*Scaphiophryne madagascariensis*	228.68
Barbourula busuangensis	524.14	*Guibemantis liber*	142.62	*Nectophrynoides viviparus*	235.83	*Scaphiopus holbrookii*	356.82
Barycholos pulcher	156.99	*Haddadus binotatus*	276.68	*Nothophryne broadleyi*	137.18	*Semnodactylus wealii*	171.72

Batrachyla taeniata	224.17	*Hadromophryne natalensis*	385.54	*Nyctibates corrugatus*	320.78	*Sooglossus sechellensis*	131.49
Bombina maxima	277.35	*Heleioporus australiacus*	307.36	*Nyctimystes daymani*	331.46	*Spea multiplicata*	310.25
Boophis boehmei	214.74	*Heleophryne purcelli*	280.68	*Nyctixalus spinosus*	263.65	*Strabomantis ingeri*	403.44
Brachycephalus ephippium	117.35	*Hemiphractus proboscideus*	414.43	*Nyctixalus pictus*	225.82	*Stumpffia pygmaea*	68.46
Megophrys (Brachytarsophrys) carinense	662.91	*Hemisus guineensis*	151.91	*Occidozyga lima*	157.48	*Syncope antenori*	79.94
Vandijkophrynus gariepensis	350.28	*Hoplophryne uluguruensis*	96.48	*Odontobatrachus natator*	321.64	*Telmatobius laticeps*	346.74
Cacosternum namaquense	138.46	*Hyalinobatrachium fleischmanni*	130.73	*Odontophrynus americanus*	376.61	*Telmatobius degener*	284.82
Callulina kisiwamsitu	270.94	*Hypsiboas boans*	522.28	*Oreobates quixensis*	304.03	*Thaumastosaurus gezei*	473.83
Capensibufo sp.	171.18	*Ecnomiohyla tuberculosa*	484.74	*Osteocephalus buckleyi*	375.14	*Theloderma stellatum*	228.62
Cardioglossa elegans	173.11	*Hylodes perplicatus*	240.19	*Osteopilus septentrionalis*	433.96	*Thoropa miliaris*	408.21
Cardioglossa manengouba	188.77	*Hylorina sylvatica*	381.32	*Paracassina obscura*	204.94	*Tomopterna marmorata*	276.74
Calyptocephalella gayi	410.48	*Hyperolius viridiflavus*	200.08	*Pelobates fuscus*	338.04	*Trichobatrachus robustus*	448.59
Centrolene buckleyi	171.83	*Hypodactylus araiodactylus*	171.84	*Pelodytes caucasicus*	260.82	*Triprion petasatus*	290.47
Ceratobatrachus guentheri	553.66	*Ingerana tasanae*	244.63	*Peltophryne guentheri*	374.03	*Ramanella montana*	135.96
Ceratophrys aurita	954.11	*Incilius periglenes*	285.05	*Petropedetes palmipes*	336.07	*Xenopus laevis*	378.54
						Xenorhina sp.	229.69

Supplementary References

1. Laloy, F. *et al.* A re-interpretation of the Eocene anuran *Thaumastosaurus* based on MicroCT examination of a “mummified” specimen. *PLoS One* **8**, e74874 (2013).
2. Alcalde, L. & Basso, N. G. Old and new hypotheses about the homology of the compound bones from the cheek and otico-occipital regions of the anuran skull. *Zoology* **116**, 232–45 (2013).
3. Trueb, L. Patterns of cranial diversity among the Lissamphibia. in *J. Hanken & B. K. Hall (Eds.), The skull: patterns of structural and systematic diversity* 255–338 (The University of Chicago Press, 1993).
4. Trueb, L. Bones, frogs, and evolution. in *Evolutionary biology of the anurans: contemporary research on major problems* (ed. Vial, J. L.) 65–132 (University of Missouri Press, 1973).
5. Ruiz-Monachesi, M. R., Lavilla, E. O. & Montero, R. The skull of *Phyllomedusa sauvagii* (Anura, Hylidae). *Anat. Rec.* **299**, 557–572 (2016).
