# Supplementary material for: Size, microhabitat, and loss of larval feeding drive cranial diversification in frogs
Source: Nat Commun. 2021 May 4;12:2503. doi: 10.1038/s41467-021-22792-y (PMC8096824; doi:10.1038/s41467-021-22792-y)
Supplement: Supplementary file 2 — Description of Additional Supplementary Files [file 41467_2021_22792_MOESM2_ESM.pdf]

Description of Additional Supplementary Files

File Name: Supplementary Data 1
Description: Specimen and scan details, as well as phylogenetic, developmental and ecological information for species in this study and information on the absence of cranial regions. For regions classed as absent across the specimens, 'absence' includes absolute absence of a region, as well as unossified regions. MfN: Museum für Naturkunde Berlin, Germany; MNHN: Muséum national d'histoire naturelle, Paris, France; UF: University of Florida, Florida, USA; UT: University of Texas, Austin, USA. *specimens without species assignment, so the name displayed here indicates the position we assigned these specimens for phylogenetic analyses. **specimen was mirrored as the right side was damaged or incomplete. ***reference did not specify if larvae were feeding or non-feeding, but feeding was assumed if larvae were freeliving (or this genus had feeding larvae), and non-feeding was assumed if species was a direct developer.
