# Supplementary material for: Size, microhabitat, and loss of larval feeding drive cranial diversification in frogs
Source: Nat Commun. 2021 May 4;12:2503. doi: 10.1038/s41467-021-22792-y (PMC8096824; doi:10.1038/s41467-021-22792-y)
Supplement: Supplementary file 4 — Reporting Summary [file 41467_2021_22792_MOESM4_ESM.pdf]

nature research

Corresponding author(s): Anjali Goswami
Last updated by author(s): Jan 26, 2021

Reporting Summary

Nature Research wishes to improve the reproducibility of the work that we publish. This form provides structure for consistency and transparency in reporting. For further information on Nature Research policies, see our Editorial Policies and the Editorial Policy Checklist.

Statistics

For all statistical analyses, confirm that the following items are present in the figure legend, table legend, main text, or Methods section.

n/a	Confirmed	
☐	☒	The exact sample size (n) for each experimental group/condition, given as a discrete number and unit of measurement
☐	☒	A statement on whether measurements were taken from distinct samples or whether the same sample was measured repeatedly
☐	☒	The statistical test(s) used AND whether they are one- or two-sided<br>*Only common tests should be described solely by name; describe more complex techniques in the Methods section.*
☐	☒	A description of all covariates tested
☐	☒	A description of any assumptions or corrections, such as tests of normality and adjustment for multiple comparisons
☐	☒	A full description of the statistical parameters including central tendency (e.g. means) or other basic estimates (e.g. regression coefficient) AND variation (e.g. standard deviation) or associated estimates of uncertainty (e.g. confidence intervals)
☐	☒	For null hypothesis testing, the test statistic (e.g. F, t, r) with confidence intervals, effect sizes, degrees of freedom and P value noted<br>*Give P values as exact values whenever suitable.*
☒	☐	For Bayesian analysis, information on the choice of priors and Markov chain Monte Carlo settings
☒	☐	For hierarchical and complex designs, identification of the appropriate level for tests and full reporting of outcomes
☐	☒	Estimates of effect sizes (e.g. Cohen's d, Pearson's r), indicating how they were calculated

Our web collection on statistics for biologists contains articles on many of the points above.

Software and code

Policy information about availability of computer code

Data collection	Meshes from microCT scans were created in Avizo Lite 9 (FEI Visualization Sciences Group, Burlington, MA, USA) and VG Studio MAX2 and processed in Geomagic Wrap 2017 (3D Systems, Rock Hill, South Carolina, USA) to remove noise and small surface foramina. Landmark and curve semilandmarks were collected using IDAV Landmark Editor v.3.6, and surface semilandmarks placed using the R package Morpho v.2.7 in R v3.6.1.
Data analysis	All data analysis was conducted in the R software environment with openly available software packages. Analyses were conducted in R v3.6.1 and using the following R packages: Morpho v2.7, geomorph v3.1.3, phytools 0.6-60, mvMORPH 1v.1.4, Rphylopars v0.2.11, ape v5.3.

For manuscripts utilizing custom algorithms or software that are central to the research but not yet described in published literature, software must be made available to editors and reviewers. We strongly encourage code deposition in a community repository (e.g. GitHub). See the Nature Research guidelines for submitting code & software for further information.

Data

Policy information about availability of data

All manuscripts must include a data availability statement. This statement should provide the following information, where applicable:

- Accession codes, unique identifiers, or web links for publicly available datasets
- A list of figures that have associated raw data
- A description of any restrictions on data availability

All 3D meshes are available on the online repositories Morphosource.org and phenome10k.org for use by other researchers. Morphospace DOIs for all 173 meshes are detailed in Supplementary Data File 1. Landmark and semilandmark data are available at https://github.com/anjgoswami/frogs_modularity: DOI: https:/doi.org/10.5281/zenodo.4619880.

Field-specific reporting

Please select the one below that is the best fit for your research. If you are not sure, read the appropriate sections before making your selection.

☐ Life sciences ☐ Behavioural & social sciences ☒ Ecological, evolutionary & environmental sciences

For a reference copy of the document with all sections, see nature.com/documents/nr-reporting-summary-flat.pdf

Ecological, evolutionary & environmental sciences study design

All studies must disclose on these points even when the disclosure is negative.

Study description	We used high-dimensional 3D surface semilandmark data for a broad dataset of anuran cranial specimens spanning their living diversity and applied a suite of phylogenetic comparative methods to estimate the influences of skull size, larval feeding mode, adult microhabitat, and ossification sequence on the morphology, evolutionary rate, and disparity of the anuran cranium as a whole and separately for 15 cranial regions.
Research sample	Micro-CT scans of crania for 173 anuran species were derived from specimens in the following international museum collections: MfN: Museum für Naturkunde Berlin, Germany; MNHN: Muséum national d'histoire naturelle, Paris, France; UF: University of Florida, Florida, USA; UT: University of Texas, Austin, USA. Scan information and specimen locations and numbers can be found in Supplementary Data File 1.
Sampling strategy	Our sampling strategy was to capture the full phylogenetic breadth of anurans, which we achieved by including representatives for every extant frog family.
Data collection	All data collection was conducted by Carla Bardua, including processing microCT scans, manually placing landmarks and curve semilandmarks, and patching surface semilandmarks. Ecological and developmental states were collected by Carla Bardua with assistance from David Blackburn. For morphometric data collection, 15 cranial regions were defined using 58 landmarks, 410 curve semilandmarks, and 527 surface semilandmarks, the last of which were applied to each cranium semi-automatically using the R package Morpho v.2.7. Landmarks and curve-semilandmarks were digitized in iDAV Landmark Editor v.3.6.
Timing and spatial scale	All morphometric, ecological, and developmental data were collected between September 2017 and December 2018.
Data exclusions	No data were excluded
Reproducibility	No experimental work was conducted
Randomization	Samples were allocated into groups based on ecological and developmental categories
Blinding	Not applicable

Did the study involve field work? ☐ Yes ☒ No

Reporting for specific materials, systems and methods

We require information from authors about some types of materials, experimental systems and methods used in many studies. Here, indicate whether each material, system or method listed is relevant to your study. If you are not sure if a list item applies to your research, read the appropriate section before selecting a response.

Materials & experimental systems

n/a	Involved in the study	
☒	☐	Antibodies
☒	☐	Eukaryotic cell lines
☒	☐	Palaeontology and archaeology
☐	☒	Animals and other organisms
☒	☐	Human research participants
☒	☐	Clinical data
☒	☐	Dual use research of concern

Methods

n/a	Involved in the study	
☒	☐	ChIP-seq
☒	☐	Flow cytometry
☒	☐	MRI-based neuroimaging

Animals and other organisms

Policy information about studies involving animals; ARRIVE guidelines recommended for reporting animal research

Laboratory animals	None

Wild animals	Museum specimens only, no new samples were collected for this study
Field-collected samples	Not applicable
Ethics oversight	Not applicable

Note that full information on the approval of the study protocol must also be provided in the manuscript.
